# Ameliorating the Metabolic Burden of the Co-expression of Secreted Fungal Cellulases in a High Lipid-Accumulating *Yarrowia lipolytica* Strain by Medium C/N Ratio and a Chemical Chaperone

**DOI:** 10.3389/fmicb.2018.03276

**Published:** 2019-01-09

**Authors:** Hui Wei, Wei Wang, Hal S. Alper, Qi Xu, Eric P. Knoshaug, Stefanie Van Wychen, Chien-Yuan Lin, Yonghua Luo, Stephen R. Decker, Michael E. Himmel, Min Zhang

**Affiliations:** ^1^Biosciences Center, National Renewable Energy Laboratory, Golden, CO, United States; ^2^Department of Chemical Engineering, The University of Texas at Austin, Austin, TX, United States; ^3^National Bioenergy Center, National Renewable Energy Laboratory, Golden, CO, United States

**Keywords:** fungal cellulolytic enzymes, *Yarrowia lipolytica*, cellulosic biofuel, cellobiohydrolase I, endoglucanase II, lipid metabolism, endoplasmic reticulum stress, chemical chaperone

## Abstract

*Yarrowia lipolytica*, known to accumulate lipids intracellularly, lacks the cellulolytic enzymes needed to break down solid biomass directly. This study aimed to evaluate the potential metabolic burden of expressing core cellulolytic enzymes in an engineered high lipid-accumulating strain of *Y. lipolytica*. Three fungal cellulases, *Talaromyces emersonii*-*Trichoderma reesei* chimeric cellobiohydrolase I (chimeric-CBH I), *T. reesei* cellobiohydrolase II (CBH II), and *T. reesei* endoglucanase II (EG II) were expressed using three constitutive strong promoters as a single integrative expression block in a recently engineered lipid hyper-accumulating strain of *Y. lipolytica* (HA1). In yeast extract-peptone-dextrose (YPD) medium, the resulting cellulase co-expressing transformant YL165-1 had the chimeric-CBH I, CBH II, and EG II secretion titers being 26, 17, and 132 mg L^-1^, respectively. Cellulase co-expression in YL165-1 in culture media with a moderate C/N ratio of ∼4.5 unexpectedly resulted in a nearly two-fold reduction in cellular lipid accumulation compared to the parental control strain, a sign of cellular metabolic drain. Such metabolic drain was ameliorated when grown in media with a high C/N ratio of 59 having a higher glucose utilization rate that led to approximately twofold more cell mass and threefold more lipid production per liter culture compared to parental control strain, suggesting cross-talk between cellulase and lipid production, both of which involve the endoplasmic reticulum (ER). Most importantly, we found that the chemical chaperone, trimethylamine N-oxide dihydride increased glucose utilization, cell mass and total lipid titer in the transformants, suggesting further amelioration of the metabolic drain. This is the first study examining lipid production in cellulase-expressing *Y. lipolytica* strains under various C/N ratio media and with a chemical chaperone highlighting the metabolic complexity for developing robust, cellulolytic and lipogenic yeast strains.

## Introduction

As one of the most abundant renewable resources today, lignocellulosic biomass is under development worldwide to produce biofuels and chemicals. One key bottleneck hindering biofuels development is the high cost of conversion of feedstocks to sugars due to the general recalcitrance of plant cell walls (Himmel et al., 2007). To overcome this hurdle, a process strategy of cellulase production, cell wall polymer hydrolysis, and sugar fermentation in a single step termed consolidated bioprocessing (CBP) has been proposed (Lynd et al., 2005). So far, *Saccharomyces cerevisiae* and *Kluyveromyces marxianus* (Fan et al., 2012; Olson et al., 2012; Sun et al., 2012; Chang et al., 2013; Yamada et al., 2013; Kricka et al., 2014), and recently *Yarrowia lipolytica* (Wei et al., 2014; Guo et al., 2017) have been explored as potential CBP microorganisms.

*Yarrowia lipolytica* has long been known to be oleaginous and recently, has been engineered to utilize xylose for lipid production (Ledesma-Amaro et al., 2016; Li and Alper, 2016; Markham et al., 2016; Rodriguez et al., 2016). The accumulated lipids can be extracted and upgraded for biodiesel production (Ratledge and Wynn, 2002; Sitepu et al., 2014). Because *Y. lipolytica* lacks the cellulolytic enzymes needed to break down cellulosic biomass directly (Ryu et al., 2015), efforts have been made recently to express cellulases in this yeast, specifically CBH I, CBH II, endoglucanase II (EG II), β-D-glucosidase, and xylanase (Boonvitthya et al., 2013; Wang et al., 2014a; Wei et al., 2014; Guo et al., 2015). We note that a consortium co-culture of these cellulase transformants demonstrated synergy in utilizing cellulose (Wei et al., 2014). Most recently, progress has been made in co-expressing CBHs, EGs, and BGLs in *Y. lipolytica* to mimic the ratio of the main cellulases in the secretome of *T. reesei*. The resultant strains were shown to grow efficiently on industrial cellulose pulp, which is mostly amorphous, but limited growth on recalcitrant crystalline cellulose was also noted (Guo et al., 2017).

Another recent accomplishment is the engineering of *Y. lipolytica* to achieve a high yield of lipids or other hydrocarbons; see major reviews focused on the metabolic engineering of *Y. lipolytica* in the past 5 years (Abghari and Chen, 2014; Gonçalves et al., 2014; Zhu and Jackson, 2015; Ledesma-Amaro and Nicaud, 2016; Xie, 2017; Abdel-Mawgoud et al., 2018; Carsanba et al., 2018). Notably, the mutant pex10 mfe1 leu^-^ ura^+^ DGA1 was generated as a lipid hyper-accumulator (strain HA1), which can accumulate total lipids up to ∼90% on a cell dry-weight basis (Blazeck et al., 2014).

With the overall goal of developing a CBP platform to produce lipids as a drop-in fuel precursor from cellulosic biomass feedstocks, the objectives of this study are twofold: First, to co-express and evaluate the secretion efficiency and cellulolytic functionality of fungal CBH I, CBH II, and EG II in the abovementioned *Y. lipolytica* HA1 strain. These fungal enzymes include the Te-Tr chimeric CBH I (Ilmen et al., 2011; Wei et al., 2014), *T. reesei* CBH II, and *T. reesei* endoglucanase II (EG II). For simplicity, the co-expression of chimeric CBH I-CBH II-EG II (triplet cassette of cellulases) in this study is generally referred to as CBH I-CBH II-EG II in the text. Secondly, to investigate whether the co-expression of the core cellulolytic enzymes is a metabolic burden to the high lipid-accumulating strain of *Y. lipolytica* and whether adjusting the C/N ratio of the growth medium and supplementing the medium with a chemical chaperone can alleviate this metabolic stress. This work provides a new level of rigor for CBP strain development in yeast by exploring the relationship between cellulase production and lipid accumulation.

## Materials and Methods

### Strains, Plasmids, and Culture Medium

**FIGURE 1:**
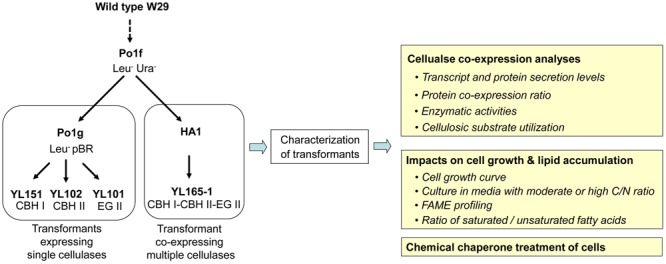
Pedigree of transformants expressing single or multiple cellulases and the experimental outline for transformant characterization. The details for these strains are described in Table 1. CBH, cellobiohydrolase; EG, endoglucanase; FAME, fatty acid methyl esters.

*Yarrowia lipolytica* strains and plasmids used in this study are described in Table 1. The pedigree of transformants expressing either a single or multiple cellulases is illustrated together with an overview of the experimental characterization of these transformants (Figure 1). Yeast strains were maintained at 28°C on YPD agar medium that contains 10 g L^-1^ yeast extract, 20 g L^-1^ peptone, 20 g L^-1^ dextrose and 15 g L^-1^ agar.

**Table 1:** *Yarrowia lipolytica* strains and plasmids.

Strain, plasmids, or constructs	Description: (strain genotype and phenotype; cloned genes and MW of encoded proteins)	Source
**Parent and derivative strains**		
Po1f	Genotype: MatA, leu2-270, ura3-302, xpr2-322, axp-2; Phenotype: Leu-, Ura-, ΔAEP, ΔAXP, Suc^+^	Madzak et al., 2000
Po1g	Genotype: MatA, leu2-270, ura3-302::URA3, xpr2-332, axp-2; Phenotype: Leu-, ΔAEP, ΔAXP, Suc^+^, pBR platform	Madzak et al., 2000
YL101	Previously named as Yl (EG II); expressing Tr eg2 (P07982; 42 kDa) with a hybrid promoter (hp4d) in parent strain Po1g.	Wei et al., 2014
YL102	Previously named as Yl (CBH II); expressing Tr cbh2 (P07987; 47 kDa) with a hybrid promoter (hp4d) in parent strain Po1g.	Wei et al., 2014
YL151	Previously named as Yl (chimeric CBH I); expressing chimeric cbh1 (Te CBH1 catalytic domain-Tr Linker-Tr CBM1; AAL89553 and P62694; 53 kDa) with a hybrid promoter (hp4d) in parent strain Po1g.	Wei et al., 2014
HA1	Genotype: Po1f Δpex10 Δmfe1 leu^-^ ura^+^ DGA1; Phenotype: prevent peroxisome biogenesis and β-oxidation by Δpex10 and Δmfe1, respectively; enhance lipid synthesis by DGA1 overexpression.	Blazeck et al., 2014
YL165-1	Genotype: Po1f Δpex10 Δmfe1 leu^-^ ura^+^ DGA1 chimeric cbh1 cbh2 eg2; Phenotype: prevent peroxisome biogenesis and β-oxidation by Δpex10 and Δmfe1, respectively; enhance lipid synthesis by DGA1 overexpression; co-express CBH I (with TEFin promoter) – CBH II (with GPD promoter) – EG II (with EXP1 promoter).	This study
**Plasmids and the cloned genes**		
pYLEX1 (i.e., pINA1269)	hybrid promoter (hp4d); selection marker gene (LEU2).	Madzak et al., 2000
pYLSC1 (i.e., pINA1296)	hybrid promoter (hp4d); secretion signal (XPR2 pre-region); selection marker gene (LEU2).	Madzak et al., 2000
pNREL151	Chimeric cbh1 (Te CBH1 catalytic domain-Tr Linker-Tr CBM1; AAL89553 and P62694; 53 kDa) in SfiI/Xbal cut pYLSC1.	Wei et al., 2014
pNREL162	Chimeric cbh1-cbh2-eg2 cassette; cloned in vector pUC57.	This work
pNREL165	Chimeric cbh1-cbh2-eg2 cassette; cloned in vector pYLEX1.	This work

*The theoretical molecular weight (MW) was calculated based on amino acid sequence (without a signal peptide). CBH, cellobiohydrolase; EG, endoglucanase; Te, Talaromyces emersonii; Tr, *T. reesei*.*

### Promoters, Signal Peptide, and Terminators for Cellulase Expression

Promoters were selected to mimic as closely as possible the optimal ratio of CBH I: CBH II: EG II of 60: 10: 30, or to the optimal ratio for CBH I: EG II of 90: 10 to achieve maximal cellulose degradation (Kallioinen et al., 2014; Wei et al., 2014). Thus the promotors TEFin, GPD, and EXP1 with expression levels of approximately 17, 0.8, and 1.2-fold that of TEF (Damude et al., 2011; Tai and Stephanopoulos, 2013) were chosen to control the expression of individual cellulases.

**FIGURE 2:**
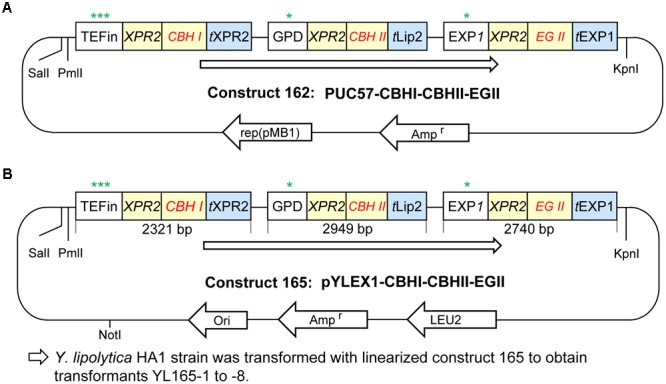
Plasmid construction for cellulase expression in *Yarrowia lipolytica*. **(A)** Plasmid construct 162 with CBH I-CBH II-EG II cloned in vector pUC57. **(B)** Plasmid construct 165 with CBH I-CBH II-EG II cloned in the *Y. lipolytica* expression vector pYLEX1, which carries Leu2 as a selection marker. More details are described in the Materials and Methods. Symbols and abbreviations: rep (pMB1), origin of replication derived from plasmid pMB1; t, terminator; ^∗, ∗∗^, and ^∗∗∗^ indicate the relatively low, middle and high expression levels of promoters, respectively.

Details for the promoters, signal peptide and terminators for expressing individual cellulases are described as below. Chimeric CBH I was expressed by the TEFin promoter and the XPR2 terminator (GenBank, accession no. M23353) (Davidow et al., 1987). CBH II was expressed by the GPD promoter (YALI0C06369p; -931 to -1; GenBank nucleotide 158467892) and the Lip2 terminator (GenBank accession no. AJ012632). EG II was expressed by the EXP1 promoter and EXP1 terminator (Damude and Zhu, 2007; Ye et al., 2012). Each of three cellulase genes contains an XPR2 signal peptide (AAGCTCGCTACCGCCTTTACTATTCTCACGGCCGTTCTGGCC) and was codon optimized according to the codon optimization table for *Y. lipolytica*. The cellulase expression cassette (i.e., CBH I-CBH II-EG II cassette; named as construct 162) was synthesized by GenScript Inc. (Piscataway, NJ, United States) with SalI-PmlI sites on the 5′ end and a KpnI site on its 3′ end (see sequence in the additional file). This expression cassette was cloned into the vector pUC57 at the SalI and EcoRV sites. The sequence of the complete expression cassette of construct 162 is provided in Supplementary Materials and Methods. Finally, to build the *Y. lipolytica* cellulase secretion construct (named as construct 165), construct 162 and vector pYLEX1 were digested with SalI and KpnI, and ligated together (Figures 2A,B).

### Transformation and Selection

Random integrative transformation of *Y. lipolytica* strain HA1 with NotI-linearized plasmid 165 DNA was conducted using YLOS One Step Transformation system and the YLEX expression kit (Yeastern Biotech Co., Taipei, Taiwan) as described previously (Wang et al., 2014a). The transformation mixture was spread on YNB selection plates lacking leucine for the appearance of transformant (Leu^+^) colonies.

### RNA Extraction and Real-Time RT-PCR

Total RNA was extracted from 60 to 80 mg (wet weight) cell pellets of transformants using Qiagen RNeasy Mini Kit (Valencia, CA, United States). The procedure for cDNA synthesis was similar to the protocol described by Wei and coworkers (Wei et al., 2012). One microgram of purified total RNA was reverse-transcribed using High-Capacity cDNA Reverse Transcription Kit (Cat. no. 4368814, Applied Biosystems, Grand Island, NY, United States) with random hexamers according to the manufacturer’s instructions.

Primers were designed for real-time RT-PCR targeting the codon-optimized sequences for individual cbh1, cbh2, and eg2 genes expressed in *Y. lipolytica* transformants (Supplementary Table S1). Primers for the reference gene encoding actin (YALI0D08272g) were previously described (Dulermo et al., 2015). Real-time RT-PCR was performed using ABI 7500 Real-Time PCR System (Thermo Fisher Scientific, Applied Biosystems, Grand Island, NY, United States) and Power SYBR Green PCR Master Mix (Cat. no. 4367659, Applied Biosystems, Grand Island, NY, United States). PCR reactions were performed in triplicate. The relative transcription level of genes was calculated from the Ct value of reference gene and gene-of-interest (Schmittgen and Livak, 2008).

### SDS–PAGE and Western Blot

The supernatant from each strain was collected from YPD pH 4.0 medium when the culture reached an OD600 value of 10. The loading amount per well was 22.5 μL mixed with 7.5 μL 4X loading buffer, following the procedures described previously for the SDS–PAGE and western blot analyses (Wei et al., 2014), for which the custom mouse monoclonal anti-CBH I, mouse monoclonal anti-CBH II, and rabbit polyclonal anti-EG II were used. Densitometric analysis of the detected bands was performed in accordance with literature (Savina et al., 2002; Olszanecki et al., 2007; Rashid et al., 2015) using the Quantity One software (Bio-Rad, CA, United States). The band intensities, relative to the corresponding bands in the protein samples of strains expressing individual cellulases, are presented as the average values from three separate experiments.

### Cellulosic Substrates for Enzymatic Activity Assays, Screening, and Culturing of Transformants

Two types of cellulosic substrates were used in this study:

(1) Phosphoric acid swollen cellulose (PASC) was prepared from Avicel PH101 (cat. no. 11365, Sigma) using the procedure described in literature (Schulein, 1997; Harris et al., 2010).
(2) Wet ball-dispersed Avicel was prepared by suspending 1% Avicel in 50 mM acetate buffer, pH 4.8 in capped Fisherbrand glass bottles containing sterile glass beads with an average diameter of 0.7 mm and shaking overnight at 180 rpm and 25°C; the volume ratio of 1% Avicel: glass beads was 5:1, with the volume of glass beads being measured by sterile cylinder. Note that proper dispersion of Avicel is important for testing the optimal functionality of cellulases secreted by transformants. Wet ball milling has been reported to decrease the particle size and increase the surface area of cellulose (Ishikawa and Ide, 1993; Kobayashi et al., 2011) and is often used for particle dispersion of various materials (Hussain et al., 1996; Munkhbayar et al., 2013).

### Screening CBH I-CBH II-EG II Co-expressing Transformants on YPD-PASC Plates

The capacity of transformants to utilize cellulose was assessed by clearing zones on 0.5% PASC-YPD agar plates (0.5% PASC, 1.0% yeast extract, 2.0% peptone, 2.0% dextrose, 1.5% agar). The plates were inoculated with strains and then incubated at 28°C for 6 days before Congo Red staining (Wood and Mahalingeshwara Bhat, 1988).

### Enzyme Activity Assay

The combined enzymatic activity of co-expressed CBH I-CBH II-EG II was measured by using unconcentrated or concentrated supernatants of yeast cell cultures. The supernatants were collected from YPD cultures after 5 days shaking at 30°C, 200 rpm. The concentrated crude enzymes were prepared by concentrating 35-fold using ultrafiltration with a molecular weight cut-off of 10,000-dalton. For enzyme activity measurements, 0.5 mL of the supernatants or concentrated crude enzyme solutions was mixed with 0.5 mL of 1% Avicel suspended in 50 mM acetate buffer at pH 4.8. As controls, 0.5 mL of the concentrated crude enzyme was mixed with 0.5 mL acetate buffer without the substrate; in parallel, Avicel without enzyme (using ddH_2_O instead) was also set as controls. The replicate vials containing enzyme-Avicel mixtures or controls were incubated at 50°C for 1 h, 24 h, and 5 days. The samples were centrifuged at 12,000 rpm for 3 min and the supernatant was filtered through a 0.45-μm filter. The released sugars were measured by high-performance liquid chromatography (HPLC). The “% Avicel to glucose” conversion rate was calculated as the measured “Total glucose equivalent released g L^-1^” divided by 5 g L^-1^, which was the amount of total glucose equivalent released for theoretical 100% Avicel conversion.

### Cellulose Utilization by Cellulase Co-expressing Transformants in Mineral Medium

Basal mineral media have been used to assess the cellulose utilization by filamentous fungi and yeast (Wei et al., 2013, 2014; Guo et al., 2017). The mineral medium described by Guo et al. (Guo et al., 2017) contained higher essential nutrients, and was used in this study. In addition to CBH and EG, functionally potent BGL is also needed for the conversion of cellulose to glucose (Dashtban and Qin, 2012). In general, *Y. lipolytica* is viewed as limited in endogenous ability to digest cellobiose, with some cellobiose-consuming wild-type, or substrate-adapted strains being reported (Kurtzman et al., 2011; Mirbagheri et al., 2012; Lane et al., 2015; Ryu et al., 2016). A strategy of adding exogenous BGL was used in previous studies to boost the low BGL activity in *T. reesei’s* cellulase preparations (Xin et al., 1993; Berlin et al., 2007; Chen et al., 2008). This study follows these previous examples by adding exogenous BGL to the medium so that BGL would not be a limiting factor for cellulose degradation.

Briefly, seed cell culture was grown in 20 mL YPD medium in a 125-mL baffled flask overnight at 28°C, followed by centrifugation for cell collection. The cells were washed with sterile ddH_2_O, and used to inoculate 100 mL of mineral medium in 500-mL baffled flasks to reach an initial OD of 1.6 (equivalent to approximately 1 g DCW L^-1^; DCW basis), which was comparable to the inoculation rate reported in literature (Guo et al., 2017). The mineral medium was supplemented with 2.7 g Avicel (i.e., 2.7% w/v), as well as with BGL (Aspergillus niger BGL: Cat. no. E-BGLUC, Megazyme, International Ireland Ltd., Wicklow, Ireland), at a concentration of 2 mg BGL per gram cellulose substrate based on the literature (Selig et al., 2014; Wei et al., 2014; Xu et al., 2017). The BGL used was chromatographically purified prior to use. The flasks that contained the cell-medium-Avicel-BGL mixtures were incubated in a rotary shaker at 200 rpm and 28°C for 5 days. The cells were centrifuged, freeze-dried, and subjected to Avicel residue analysis, as described below. Three biological cultures were run for the cell mixtures.

### Quantification of Avicel Residues and Cell Weight in Avicel-Yeast Cell Pellets

The determination of Avicel residues and cell dry weight was conducted as previously described (Wei et al., 2014; Guo et al., 2017). Briefly, the Avicel-yeast cells from the transformant culture growing on Avicel were centrifuged, washed with ddH_2_O, freeze-dried, and weighed. The amount of Avicel contained in the pellet was determined by enzymatic digestions using the Cellic CTec2 cellulase enzyme product (Novozyme, Franklin, NC, United States) and cross-checked by diluted acid (2.5% sulfuric acid) hydrolysis of the residues with at 121°C for 1 h. The total glucose released was measured by HPLC and taken as the corresponding amount of Avicel contained in the Avicel-yeast cell mix. Cell dry weight was calculated by subtracting the amount of Avicel from the weight of Avicel-cell yeast pellet.

### Growth Curves and Validation

Growth curves were obtained by using a Bioscreen C analyzer (Growth Curves United States, Piscataway, NJ, United States) and a modified protocol for cell inoculation, growth conditions, and turbidity measurements (Franden et al., 2013; Wang et al., 2014b). In brief, log phase cultures of *Y. lipolytica* strains were used to inoculate 20 mL YPD pH 4.0 medium (as a cellulase production medium), which had an initial C/N ratio of approximately 4.5 (Martinez-Force and Benitez, 1995; Josefsen et al., 2012), in 150 mL flask for overnight growth at 30°C and 210 rpm. After overnight growth, the culture reached an OD_600_ ∼ 10. Cells were then diluted into fresh YPD pH 4.0 medium at an initial OD600 of 0.25 and distributed into Bioscreen C microplates (three wells per cell line; 300 μL per well). Incubation for the Bioscreen C microplates was performed at 30°C for 5 days, with absorbance readings taken every 15 min. The turbidity measurement with a wide band filter (420–580 nm, which is relatively insensitive to color changes) were computer operated with EZ Experiment software. The collected data were exported to spreadsheets of Microsoft Excel, and the turbidity data was averaged from three replicates of cell samples.

Cell mass dry weight measurements in shake flask cultures were used to validate the growth curves generated using the Bioscreen C analyzer. Overnight cultures were used to inoculate 200 mL YPD pH 4.0 medium in 1000-mL baffled flask to reach an initial OD600 of 0.25. The flasks were incubated in a rotary shaker at 200 rpm and 30°C for 5 days, during which ten milliliters of the cell cultures were collected after 6, 12, 24, 48, 72, 96, and 120 h and centrifuged at 4000 g for 5 min. The collected cell pellets were washed three times with sterile distilled-deionized water (ddH_2_O) to remove medium residues and collected by centrifugation at 4000 g for 5 min. The cell pellets obtained were freeze dried and weighed. Cell weight data were averaged from three replicate samples.

### Glucose Consumption and Lipid Accumulation in Moderate and High C/N Media

Two types of media were used to grow the cellulase co-expressing transformants for investigating their glucose consumption and lipid accumulation. One was a moderate C/N ratio medium prepared from the YPD medium supplemented with 10 g L^-1^ extra glucose (with the final glucose of 30 g L^-1^, thus referred as YPD-3% Glu), with unadjusted pH and a C/N ratio > 4.5 (Martinez-Force and Benitez, 1995; Josefsen et al., 2012). Another was a high C/N ratio medium adapted from literature (Blazeck et al., 2014), which contained 80 g L^-1^ glucose, 6.7 g L^-1^ Yeast Nitrogen Base w/o amino acids (containing 5 g L^-1^ ammonium sulfate, which corresponds to 1.365 g L^-1^ ammonium, or approximately 38 mM nitrogen), and 0.79 g L^-1^ CSM supplement (Cat. no. 114500012; MP Biomedicals). The C/N ratio (g/g) of this high C/N ratio medium was approximately 59, with a pH value of 4.7. Both media were sterilized by 0.2-μm filtration.

Briefly, an overnight growth of transformants and the parent control strains grown in YPD medium was used to inoculate 50 mL of either YPD-3% Glu medium or the high C/N ratio medium in 250-mL baffled flasks to reach an initial OD of 0.25. The flasks were incubated in a rotary shaker at 200 rpm and 28°C for 6 days, followed by centrifugation for cell harvest. The cell pellets were freeze-dried and subjected to FAME analysis, as described previously (Wei et al., 2013).

### Treatment of Yeast Cells With a Chemical Chaperone

The chemical chaperone, trimethylamine N-oxide dihydride (TMAO; Cat. no T0514, Sigma), was used to examine its effect on the cell growth and lipid accumulation of *Y. lipolytica* control strain and the cellulase-expressing transformants. A stock solution of 3 M TMAO was prepared by dissolving the chemical in the culture medium and sterilized by using 0.22 μm filter.

Briefly, an overnight growth of transformants and the parent control strains grown in YPD was used to inoculate 50 mL of high C/N ratio medium in 250-mL baffled flasks to reach an initial OD of 0.25. The flasks were incubated in a rotary shaker at 200 rpm and 28°C for 4 days, followed by adding 2.63 mL of sterile 3 M TMAO stock solution to reach a working concentration of 150 mM. The flasks were incubated in a rotary shaker at 200 rpm and 28°C for another 2 days, then centrifuged for cell harvest. The dose and duration of the chemical chaperone treatment were based on literature (Thanonkeo et al., 2007; Sootsuwan et al., 2013; Lamont and Sargent, 2017). Untreated parallel cultures (without TMAO) were used as controls. The cell pellets were freeze-dried and subjected to FAME analysis, as described previously (Wei et al., 2013).

## Results and Discussion

### Co-expression of CBH I-CBH II-EG II in *Y. lipolytica* HA1

Prior to the present work, the enzymes chimeric CBH I, CBH II, and EG II had been expressed in *Y. lipolytica* individually (Wei et al., 2014). To co-express these three enzymes, plasmid construct 165 was built in the backbone of pYLEX1 vector, and the resultant plasmid was named as pYLEX1-CBH I-CBH II-EG II with LEU2 as selection marker (Figure 2). The plasmid construct 165 was linearized with NotI and transformed into *Y. lipolytica* HA1.

Approximately 2000 Leu^+^ transformants were recovered on YNB plates lacking leucine. Since the cells we used for transformation were not synchronized, they likely were in different stages of their cell cycle. In addition, random insertion-associated positional effects have the potential to lead to a range of size variation among the resulted positive colonies of transformants on the selection plates. Out of these transformants, 20 visually larger colonies were selected as, in general, the large colonies are more likely to be stable transformants from transformation of *Y. lipolytica* (Orr-Weaver and Szostak, 1983) and colony size has been used as a proxy for fitness in yeast and other microorganisms (Baryshnikova et al., 2010; Wagih et al., 2013). These 20 transformants were further narrowed down to eight transformants based on their higher OD600 values in YNB liquid medium after overnight growth. The single-colony purified transformants were tested for their ability to hydrolyze cellulose by growth on PASC-YPD agar for 6 days followed by Congo Red staining. The results showed that five out of the eight transformants (YL165-1, YL165-5, YL165-6, YL165-7, and YL165-8) produced relatively large clearance zones on the PASC-YPD plates after Congo Red staining (Supplementary Figures S1A,B). These transformants were confirmed to express CBH I-CBH II-EG II by real-time RT-PCR (Supplementary Figure S2).

It is noteworthy that in this study, two colony sizes appeared on the selection plates: large colonies appeared at day 3 after plating while smaller became visible at day 5 to 6 after plating. In addition to the picking of 20 larger colonies as described above, we also picked 20 of the later smaller colonies. However, further culturing of the smaller colonies indicated that all of them were false positive as they did not grow after re-streaking on selection plates, which was not an unusual phenomenon in fungal and yeast transformation caused by transient expression or unstable insertion of target genes and marker into the host genome (Singh A. et al., 2015; Schwarzhans et al., 2016).

### Enzyme Activity Screening of Transformants Co-expressing CBH I-CBH II-EG II

Further investigation was conducted to measure the enzyme activity of the culture supernatant or concentrated crude enzymes using wet ball-dispersed Avicel as a substrate. Enzymatic conversion of Avicel to cellobiose and glucose was measured after 1 h, 24 h, and 5 days digestion. For unconcentrated supernatants of transformants of YL165-1, YL165-5, and YL165-7, the enzyme activities (expressed as released sugars) were only measurable after 24 h and 5 days of hydrolysis, with up to 11% of Avicel being converted to cellobiose and glucose (Table 2, columns 3–4). For transformants YL165-6 and YL165-8, the enzyme activities of their unconcentrated supernatants were the lowest, only converting 3–4% of Avicel to cellobiose and glucose after 5 days of incubation with the Avicel substrates. Thus, these transformants were eliminated from further testing of their supernatants for enzymatic analysis.

**Table 2:** Enzyme activity of supernatant or concentrated crude enzymes of transformants co-expressing CBH I-CBH II-EG II.

Enzyme-Avicel incubation time (50°C)	*Y. lipolytica* strain	1× supernatant enzyme activity		35× concentrated crude enzyme activity	
		Total Glu equiv. released g L^-1^	% Avicel to Glu equiv.	Total Glu equiv. released g L^-1^	% Avicel to Glu equiv.
0 h	HA1 (EV)	0	0	0	0
	YL165-1	0	0	0	0
	YL165-5	0	0	0	0
	YL165-7	0	0	0	0
1 h	HA1 (EV)	0	0	0	1%
	YL165-1	0	0	1.56	31%
	YL165-5	0	0	1.19	24%
	YL165-7	0	0	0.63	13%
24 h	HA1 (EV)	0	0	0.04	1%
	YL165-1	0.30	6%	2.78	56%
	YL165-5	0.24	5%	2.48	50%
	YL165-7	0.14	3%	1.34	27%
5 days	HA1 (EV)	0.00	0%	0.11	2%
	YL165-1	0.53	11%	3.45	69%
	YL165-5	0.50	10%	3.28	66%
	YL165-7	0.26	5%	1.55	31%

*Data presented were the average of three biological replicates and the SEM (standard error of the mean) was less than 10%. Glu equiv., glucose equivalent. HA1 (EV), parent control strain HA1 transformed with empty vector.*

With the above low percent conversion in the culture supernatants and to better understand the potential enzymatic activity present in these culture supernatants, the culture supernatants of YL165-1, YL165-5, and YL165-7 were concentrated 35×. In the concentrated supernatants, significant conversion of Avicel, 13% to 31%, was observed after 1 h. Conversion increased to 56 and 69% for transformant YL165-1 and 50% and 66% for YL165-5 after 24 h and 5 days, respectively (Table 2, columns 5–6), indicating that 35x concentrated cellulases can significantly enhance the degradation of cellulose.

Microscopic imaging analysis of the Avicel-crude enzyme mixture after 5 days incubation with 35x concentrated crude enzymes of transformant YL165-1 confirmed the effects of cellulases on the size of Avicel particles. Compared to Avicel granules incubated with no enzymes, the particles of Avicel incubated with 35x concentrated crude enzymes of transformant YL165-1 were found to be much finer (Supplementary Figure S3), confirming deconstruction of the crystalline cellulose particles.

### Comparing Cellulase Levels in YL165-1 vs. Single Cellulase Expressing Strains

**FIGURE 3:**
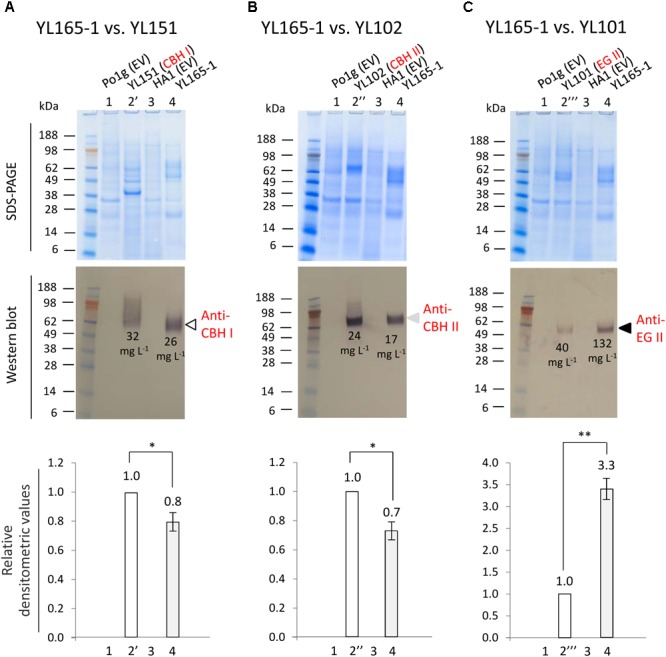
Comparison of cellulase secretion levels between CBH I-CBH II-EG II co-expressing *Y. lipolytica* transformant YL165-1 and the individual, single cellulase expressing transformants. **(A)** Supernatant samples for western blot using anti-Tr CBH I antibody. **(B)** Supernatant samples for western blot using anti-CBH II antibody. **(C)** Supernatant samples for western blot using anti-EG II antibody. In **(A–C)**, the upper panels show SDS–PAGE gels after staining, and the middle panels show the identically loaded gels used for the western blot, while the bottom panels show the densitometric analysis of the western blots, for which error bars indicate the SEM for three biological replicates; ^∗^ and ^∗∗^ indicate significantly different from the reference strains (that expressing single CBH I, CBH II, or EG II) with *p* < 0.05 and *p* < 0.01, respectively. Lane 1, strain Po1g (transformed with empty vector) as the parent strain control for YL151, YL102, and YL101. Lane 2′, YL151 expressing chimeric CBH I; lane 2″, YL102 expressing CBH II; lane 2″′, YL101 expressing EG II. Lane 3, strain HA1 (transformed with empty vector). Lane 4, YL165-1. Loading amount was 22.5 μL supernatant per well. Cellulase titers (g L^-1^) are indicated by numbers in the western blot images.

The best performing transformant from those described above in converting Avicel was YL165-1, which was further subjected to detailed analysis for the co-expressed cellulases. Strains YL151, YL102, and YL101, which were previously generated to express individual chimeric CBH I, CBH II, and EG II cellulases (Wei et al., 2014), were used as reference (see Figure 1 for the strain pedigree). Experimentally, these strains were arranged into three subsets and cultured in parallel in YPD media for 5 days. The supernatants were collected from the cultures when the OD600 value reached 10, followed by PAGE and western blot analyses (Figures 3A–C).

A western blot using anti-Tr CBH I antibody, which recognizes the *T. reesei* CBM and linker in the chimeric CBH I, detected a single band with the expected size for chimeric CBH I (Figure 3A, middle panel, lane 4), confirming a successful expression of chimeric CBH I in transformant YL165-1, with a protein titer being 0.8-fold of that for transformant YL151 expressing the single chimeric CBH I (Figure 3A, bottom panel, lanes 4 vs. 2′). The western blot using anti-CBH II antibody also showed a single band of the size expected for CBH II (Figure 3B, middle panel, lane 4), indicating the expression of CBH II in transformant YL165-1, with a titer being 0.7-fold of that for transformant YL102 expressing single CBH II (Figure 3B, bottom panel, lanes 4 vs. 2″). The western blot using anti-EG II antibody detected a single band with the expected size for EG II (Figure 3C, middle panel, lane 4), validating the successful expression of EG II in transformant YL165-1, with a titer being 3.3-fold of that in transformant YL101 expressing single EG II (Figure 3C, bottom panel, lanes 4 vs. 2″′).

It is noteworthy that recombinant CBH I enzymes in yeast were reported to exhibit variable levels of glycosylation, for which hyper-glycosylation leads to smeared bands in SDS–PAGE imaging (Godbole et al., 1999; Boer et al., 2000; Den Haan et al., 2007; Ilmen et al., 2011). The smeared band pattern of TeTrCBH I in YL151 (Figure 3A) was consistent with our observation in another recent study, which showed that when TeTrCBH I was expressed in the yeast *L. starkeyi*, it had a relatively high magnitude of glycosylation that led to apparently smeared band of purified TeTrCBH I band by SDS–PAGE analysis (Xu et al., 2017). Our observation here further showed that the TeTrCBH I co-expressed in YL165-1 vs. YL151 had different extents of glycosylation, as reflected by the different western blot bands in their respective lanes in Figure 3A.

### Low Correlation Between Transcriptional and Protein Levels of Cellulase Genes: Intrinsic Nature of Individual Cellulases Affecting Their Co-expression Ratio

The above data permitted a quantitation of the co-expressed cellulases in YL165-1. Previously, the titers of single chimeric CBH I, CBH II, and EG II proteins expressed were estimated to be 32, 24, and 40 mg L^-1^ in strains YL151, YL102, and YL101, respectively, in flask cultures (Wei et al., 2014). Accordingly, the titers of co-expressed CBH I, CBH II, and EG II in YL165-1 can be calculated by multiplying each single protein’s titer (in YL151, YL102, and YL101) with its respective densitometric fold-changes (Figures 3A–C, bottom panels). This gives a protein titer ratio of:

(1) $$\begin{array}{c} Protein CBH I : CBH II : EG II = 26:17:132{mg L}^{-}{}^{1}\\ = 1.0 : 0.7 : 5.1 \end{array}$$

The total titer of these cellulases is 175 mg L^-1^. Among them, EG II appears to be highly efficient for synthesis and secretion as it was the dominant cellulase based on its titer (132 mg L^-1^); in contrast, CBH I appears to be less efficient for synthesis and secretion in the obtained transformant (with a titer of 26 mg L^-1^).

Meanwhile, the real-time RT PCR analysis of cDNA samples revealed that:

(2) $$\begin{array}{c} Transcript CBH I:CBH II:EG II=15.6:1:1.4 \end{array}$$

**FIGURE 4:**
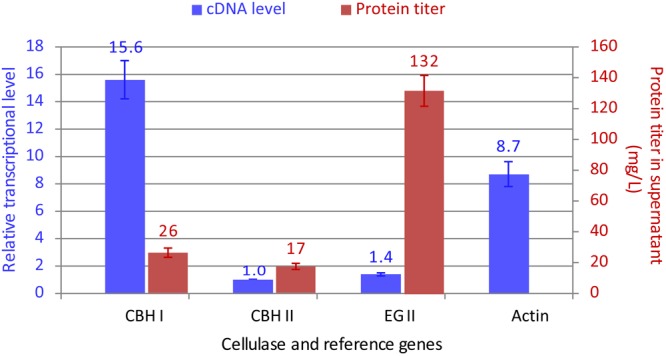
Comparison between the transcript and protein secretion levels for CBH I-CBH II-EG II co-expressed in *Y. lipolytica* transformant YL165-1. For the relative transcriptional data (in blue color), the transcriptional level for CBH II-encoding gene had the lowest mRNA level and was set at 1. The presented data were collected from three biological replicates, for which error bars indicate the SEM.

To obtain a direct, visual illustration for the transcriptional and protein levels of these cellulases, the data in Eqns. 1 and 2 were re-plotted in Figure 4, which reveals a substantial discrepancy (i.e., lack of correlation) between the transcriptional and protein levels for chimeric CBH I vs. EG II. Future studies on the co-secretion of chimeric CBH I and EG II under the same promoters are warranted.

An optimal ratio of CBH I, CBH II, and EG II is crucial for an efficient conversion of cellulose to simple sugars. This study achieved a high titer for EG II but a moderate titer for chimeric CBH I, which indicates that further efforts are needed to boost the expression level of CBH I for an optimal CBH I and EG II ratio. CBH I proteins from different fungal species vary in their expression levels and specific activity when expressed in the yeast *S. cerevisiae* (Ilmen et al., 2011) and *Y. lipolytica* (Wei et al., 2014; Guo et al., 2017). While the Te-Tr chimeric CBH I showed a likely intrinsic property for its expression limitation (26 mg L^-1^ under the control of TEFin promoter, which is a TEF promoter combined with its intron) in this study, a higher expression level of CBH I from *Neurospora crassa* (24 and 95 mg L^-1^ under control of TEF and a hybrid promoter HTEF, respectively) (Guo et al., 2017) raises hope for further tuning the ratio of CBH I and EG II by including *N. crassa* CBH I into this platform in future studies.

### Cellulose Utilization and Lipid Production by Transformant YL165-1 in Avicel Medium

The capability of *Y. lipolytica* transformant YL165-1 for utilizing cellulose and producing lipids was evaluated by growing the transformant in mineral medium with Avicel as a carbon source. The cultures were harvested at 120 h and the Avicel residues were analyzed. The results showed that YL165-1 consumed 22.8% of the original Avicel content and produced 0.28 g DCW cell biomass per g Avicel consumed (Table 3), while the control strain HA1 (EV) showed no detectable cell growth. Note that the Avicel utilization rate of 22.8% by YL165-1 is on the low end of that observed for *Y. lipolytica* strains expressing a different set of cellulase enzymes (in the range of 22–30%) (Guo et al., 2017), which can be explained by a suboptimal CBH I/EG II titer ratio of the expressed enzymes in this study.

**Table 3:** Cell mass and FAME analyses of *Y. lipolytica* transformant YL165-1 grown on mineral medium containing Avicel as sole carbon sources.

		Cell mass			FAME		
Strain	Avicel consumed %[1]	Total DCW[2]	DCW of newly grown cells[3]	Yield[4]	Total newly formed FAME[5]	FAME%	FAME yield
		g L^-1^	g L^-1^	g g^-1^ Avicel consumed	g L^-1^	DCW basis	mg g^-1^ Avicel consumed
YL165-1	22.8 ± 0.56	2.7 ± 0.12	1.7 ± 0.12	0.28 ± 0.02	0.19 ± 0.02	10.2% ± 0.7%	31 ± 3

*Data presented as the average of triplicate biological samples with SEM. DCW, dry cell weight. [1] Avicel consumed % was measured described in Methods section; The initial concentration of cellulose in the medium was 27 g L^-*1*^. [2] Total DCW was calculated as Dry weight of cell-Avicel pellet – Amount of Avicel consumed. [3] DCW of newly grown cells = Total DCW – Dry weight of initial inoculated cells; the dry weight of initial inoculated cells was 1 g L^-*1*^. [4] Cell mass yield was calculated was as g dry weight of newly grown cells per g Avicel consumed. [5] “Total newly formed FAME” = “FAME of total cells” – “FAME of initial inoculated cells.”*

Furthermore, FAME analysis showed that the total FAME produced by transformant YL165-1 was 0.19 g L^-1^, while the FAME yield was 31 mg g^-1^ Avicel consumed (Table 3). Compared with lipid production by other oleaginous microorganisms on cellulose or glucose medium, this FAME yield by YL165-1 is in a similar range. For example, it was reported that the FAME yield of the oleaginous, filamentous fungus *Mucor circinelloides* was 57 mg FAME per g Avicel consumed when supplemented with exogenous CBH I and cultured on Avicel substrates (with 33% Avicel being utilized) (Wei et al., 2013). Another report showed the yield of total lipids (which are usually higher than FAME) of the oleaginous yeast *Rhodosporidium toruloides* was 80 mg lipids per g glucose consumed when cultured in a medium (C/N ratio of 5) using glucose as substrates (Andrade et al., 2012). Note that the FAME profile of YL165-1 in Avicel medium is presented and discussed a later section. Taken together, the transformant YL165-1 was able to maintain a considerable capability for lipid accumulation using cellulose-containing medium, while the cellulose conversion efficiency remains as the limiting factor and as a future direction for strain engineering.

### Impacts of Cellulase Expression on the Growth of Transformants in YPD Medium

**FIGURE 5:**
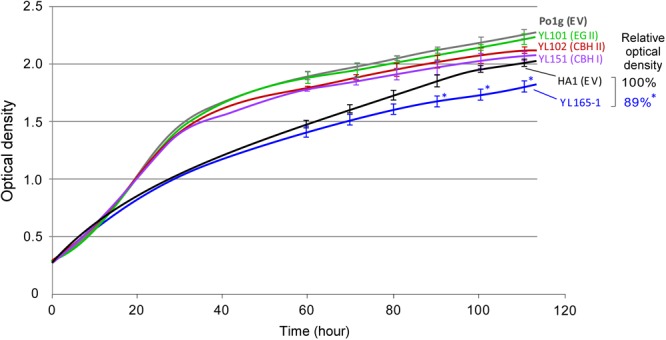
Comparison of growth curves between *Y. lipolytica* transformants expressing individual or multiple cellulases grown in YPD medium. Turbidity data were obtained from Bioscreen C, by which absorbance readings were taken every 15 min. The relative turbidity percentage values for HA1 (EV) and YL165-1 were calculated from the turbidity of the strain cultures at the end-point of the growth curve, with the turbidity of HA1 (EV) being set as 100%. HA1 (EV), strain HA1 transformed with empty vector; Po1g (EV), strain Po1g transformed with empty vector. The bars for SEM from triplicates at time points of 60, 70, 80, 90, 100, and 110 h were shown; ^∗^ indicates significantly different from the HA1 (EV) strain with *p* < 0.05.

The effects of simultaneous expression of multiple cellulases on the growth of *Y. lipolytica* was investigated by plotting the turbidity obtained from a Bioscreen C growth assay. Previously, the Bioscreen C instrument has been used to monitor microbial growth curve in terms of optical density of bacteria (Franden et al., 2009, 2013; Wang et al., 2014b) and yeast (Bom et al., 2001; Jung et al., 2015; Gientka et al., 2016). *Y. lipolytica* transformants expressing single cellulases were similar to their parent strain Po1g (EV) during the exponential growth phase and only show slight differences during the late growth phase (Figure 5). The growth curve of YL165-1 was similar to its parent control strain HA1 (EV) during exponential growth and was only 11% lower in optical density during the late growth phase (*p* < 0.05). Meanwhile, there was no significant difference between YL165-1 and HA1 (EV) in the mean size of cells based on the measurement by the Cellometer Vision (Nexcelom Bioscience, Lawrence, MA, United States). These data indicate that the co-expression of CBH I-CBH II-EG II comes at a small cost for the cells in terms of slightly reduced growth rate in acquiring the capacity to degrade and utilize cellulosic substrates.

The Bioscreen C has been used to determine the growth curve of *Y. lipolytica* in recent years (Lazar et al., 2011; Dobrowolski et al., 2016; Mirończuk et al., 2016, 2017; Rzechonek et al., 2018). Under the culture condition we used for Bioscreen C analysis, relatively a small proportion of cells changed from typical yeast form to filamentous form (as examined using Cellometer), which was not significant enough to disrupt the turbidity readings. Nevertheless, a cell dry weight method for shake-flask culture was used to validate the results of growth curve obtained via the Bioscreen C, and the data are presented in the Supplementary Figure S4. The results indicate that the timeline profiling of cell mass weight of the parent control strains and cellulase-expressing transformants follows a similar trend as their growth curves illustrated in Figure 5, confirming that the co-expression of CBH I-CBH II-EG II in YL165-1 comes at a small cost (i.e., mild metabolic burden) for the cells in terms of slightly reduced growth rate the late growth phase.

This relatively mild metabolic burden of cellulase co-expression on the growth of *Y. lipolytica* is in contrast to the relatively larger burden of cellulase expression on growth of *S. cerevisiae*, by which CBH expressing *S. cerevisiae* Y294[Y118p] strain had a 1.4-fold lower maximum specific growth rate than the reference strain (van Rensburg et al., 2012).

### Effects of Cellulase Co-expression on Lipid Production: Metabolic Drain and Its Alleviation

#### Scenario 1. Drain of Cellulase Co-expression on Lipid Production of Cells in Medium With Moderate C/N Ratio

The purpose of using a moderate C/N ratio medium (YPD-3% Glu, with a C/N ratio > 4.5) was to investigate whether cellulase co-expression causes a drain on lipid production in carbon-nitrogen balanced medium. After 6 days of culturing, most glucose in the medium was consumed by both the parent control strain HA1 (EV) and the transformant YL165-1 cells (Table 4, rows 1–2). Consistent with our above observation that the turbidity of YL165-1 cells was only slightly lower than its parent control strain HA1 (EV) at the late growth phase, the cell mass yield of YL165-1 was only slightly lower than that of HA1 (EV) (10.5 vs. 11.9 DCW g L^-1^; Table 4).

**Table 4:** Cell mass and FAME content in *Y. lipolytica* transformant YL165-1 cells cultured in moderate and high C/N media with or without a chemical chaperone.

Row no.	Media and strains	Glucose	Cell mass			FAME		
		Consumed	OD600	DCW	Yield	FAME%	Total	Yield
		g L^-1^		g L^-1^	g g^-1^ sugar	DCW basis	amount g L^-1^	mg g^-1^ sugar
	**Moderate C/N medium for FAME production at basal level**							
1	HA1 (EV)	29.2 ± 0.3	21.2 ± 0.7	11.9 ± 0.5	0.41 ± 0.02	27% ± 2%	3.2 ± 0.2	113 ± 9
2	YL165-1	29.3 ± 0.2	19.4 ± 0.8	10.5 ± 0.4^∗^	0.36 ± 0.02^∗^	15% ± 2%^∗∗^	1.5 ± 0.1^∗∗^	52 ± 6^∗∗^
	(Statistical analysis: Row 2 vs. row 1)							
	**High C/N ratio medium for FAME production at suboptimal level**							
3	HA1 (EV)	12.6 ± 0.9	5.1 ± 0.2	3.1 ± 0.1	0.25 ± 0.02	28% ± 1%	0.9 ± 0.1	69 ± 7
4	YL165-1	48.0 ± 1.3^∗∗^	16.2 ± 0.6	10.1 ± 0.3	0.21 ± 0.01	34% ± 2%^∗^	3.4 ± 0.2^∗∗^	71 ± 4^∗∗^
	(Statistical analysis: Row 4 vs. row 2)							
	**High C/N ratio medium + TMAO for FAME production at optimal level**							
5	HA1 (EV)	16.1 ± 1.1	6.2 ± 0. 2	4.1 ± 0.3	0.26 ± 0.03	24% ± 1%	1.0 ± 0.1	63 ± 8
6	YL165-1	79.9 ± 0.1^∗∗^	35.3 ± 1.2^∗∗^	21.9 ± 0.5^∗∗^	0.27 ± 0.01^∗^	27% ± 2%	5.9 ± 0.3^∗∗^	74 ± 5
	(Statistical analysis: Row 6 vs. row 4)							

*Data presented as the average of 3 biological replicate samples with SEM. ^∗^ and ^∗∗^ indicate statistical significance of *p* < 0.05 and *p* < 0.01, respectively. DCW, dry cell weight; Glu, glucose. HA1 (EV), parent control strain HA1 transformed with empty vector; TMAO, trim ethylamine N-oxide dehydrate.*

However, the FAME content in transformant YL165-1 was significantly lower than that in parent control strain HA1 (EV) (15 vs. 27% FAME, cell dry weight basis; Table 4, rows 1–2). Accordingly, the FAME yield of YL165-1 was half of that in HA1 (EV) (52 vs. 113 mg FAME per g sugar; Table 4), suggesting that co-expression of CBH I-CBH II-EG II can compromise lipid accumulation when cultured in carbon-nitrogen balanced medium.

**FIGURE 6:**
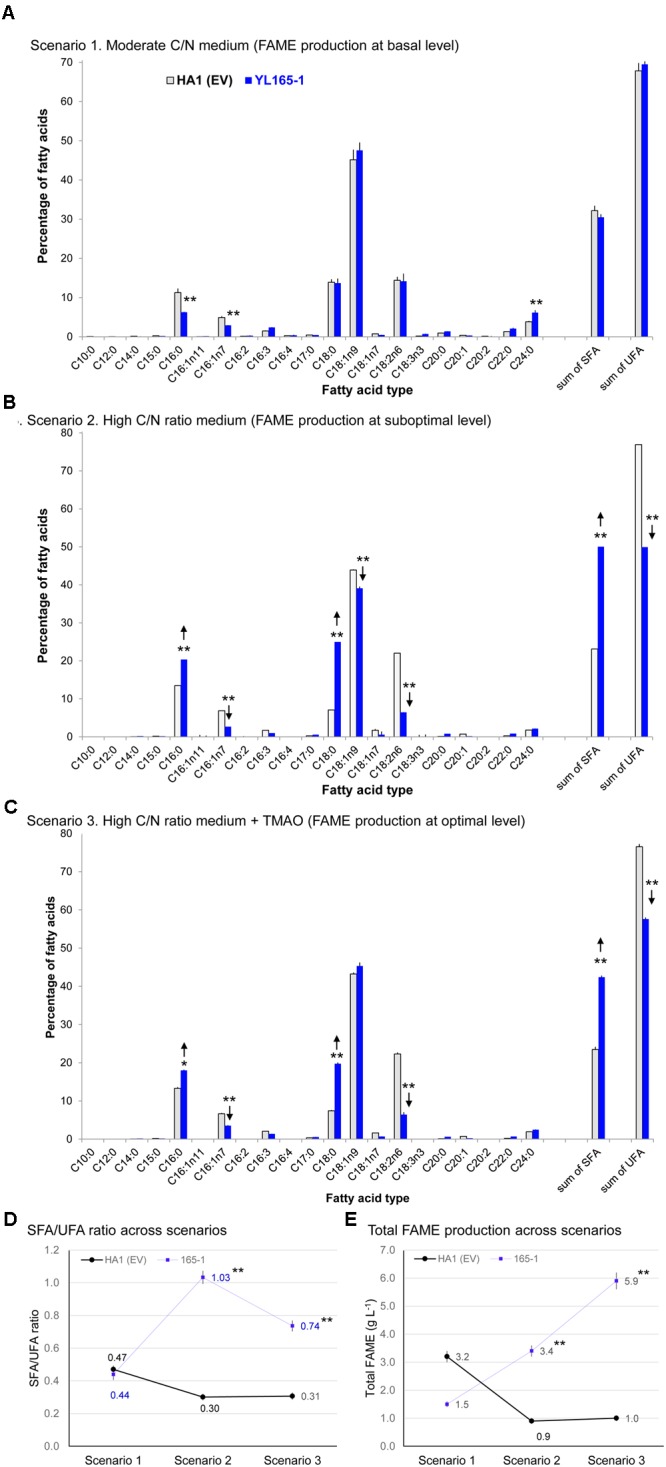
Fatty acid profiles of *Y. lipolytica* YL165-1 vs. parent control strain HA1 (EV). **(A)** Cells cultured in YPD-3% glucose medium; **(B)** cells cultured in high C/N ratio medium; **(C)** cells cultured in high C/N ratio medium supplemented with the chemical chaperone TMAO. Data presented were the mean of three replicate measurements ± SEM. ^∗^ and ^∗∗^ indicate statistical significance of *p* < 0.05 and *p* < 0.01, respectively, comparing the same fatty acid type between YL165-1 and parent control strain HA1 (EV) for **(A–C)**, or comparing the SFA/UFA ratio **(D)** or lipid production **(E)** of the same cell lines in scenarios 2 or 3 against those in scenario 1. HA1 (EV), parent strain HA1 transformed with empty vector; SFA, saturated fatty acids; SFA/UFA, ratio of saturated fatty acids and unsaturated fatty acids; TMAO, trimethylamine N-oxide dihydride; UFA, unsaturated fatty acids.

Fatty acid methyl esters analyses show that the major types of fatty acids in both types of cells were C18:1n9, C18:0, C18:2n6, and C16:0 (in order of prominence) with relatively moderate contributions from C16:1n7, C24:0, and C22:0 (Figure 6A). Such a fatty acid pattern is not only consistent with recent observation for the parent strain HA1 in which C18:1, C18:0, and C16:0 being the predominant types (Blazeck et al., 2014), but also consistent with the reported very long-chain fatty acids (VLCFA) content in the wild-type (Katre et al., 2012; Pomraning et al., 2015) and recombinant *Y. lipolytica* strains (Faure et al., 2010; Tsigie et al., 2012; Zhang et al., 2013). Overall, there is no dramatic difference between YL165-1 and HA1 (EV) in their FAME profiling despite a slight difference in the ratio of saturated fatty acids (SFA) vs. unsaturated fatty acids (UFA) (Figures 6A,D).

#### Scenario 2. Improved Lipid Production in Cellulase Co-expressing Cells by High C/N Ratio Medium

The lipid production capability of *Y. lipolytica* YL165-1 was also examined in a high C/N ratio medium, with an initial concentration of 80 g L^-1^ glucose and an initial C/N ratio of 59. For glucose consumption, after 6 days of culturing, the control strain HA1 (EV) and transformant YL165-1 cells consumed 12.6 and 48.0 g L^-1^ glucose in the medium, respectively (Table 4, rows 3–4). The control strain HA1 (EV) had a relative low OD600 of 5.1 but a high % FAME of 28% (DCW basis) (Table 4), which is consistent with previous reports showing that while a high C/N ratio medium benefited lipid accumulation, it limited the cell growth of *Y. lipolytica* (Back et al., 2016; Yang et al., 2016).

In contrast, cellulase co-expressing transformant YL165-1 grown in high C/N ratio medium showed a completely different pattern of enhanced glucose utilization (i.e., 48.0 g L^-1^ vs. 12.6 glucose g L^-1^ for the control strain). This led to twofold improvement in cell mass and a threefold increase in total FAME amount in the control strain (i.e., 10.1 vs. 3.1 g DCW, and 3.4 vs. 0.9 g FAME L^-1^ in control strain) (Table 4, rows 3–4).

Significant shifts between SFA and UFA were found in transformant YL165-1, with significantly more SFA (C16:0 and C18:0) but much less UFA (C18:1n9 and C18:2n6; Figure 6B), leading to a higher SFA/UFA ratio of 1.03 compared with the control strain’s SFA/UFA ratio of 0.30 (Figure 6D). These data suggest that cellulase co-expression can alter not only the amount but also the composition of the accumulated lipids. Our observation of increased SFA in YL165-1 grown in high C/N ratio medium is consistent with report that in the green alga *Ankistrodesmus falcatus* grown under combined stress conditions of nutrients (N, P, and Fe), both saturated fatty acid and lipid accumulation were significantly enhanced (Singh P. et al., 2015). The underlying mechanism were that under the stress conditions, unsaturated fatty acids tend to undergo oxidative cleavage, which led to higher saturated fatty acids providing oxidative stability (Sitepu et al., 2013; Singh et al., 2016).

#### Scenario 3. Lipid Production of Cellulase Co-expressing Cells Was Further Enhanced by the Addition of a Chemical Chaperone

It has been reported that chemical chaperones, a group of small-molecular-weight compounds, stabilize the conformation and structure of proteins, enhance the protein folding capacity of the ER, and help the trafficking of mutated proteins (Vallejo-Becerra et al., 2008). They are capable of reducing ER and oxidative stresses (Sootsuwan et al., 2013; Lamont and Sargent, 2017). Accordingly, we treated cellulase co-expressing transformants and control cells with the chemical chaperone TMAO, and compared the results with those from untreated cells.

The TMAO-facilitated lipid production capability of *Y. lipolytica* YL165-1 was examined in its day 4 culture grown with a high C/N ratio medium. The day 4 cells were treated with 150 mM TMAO for two additional days. For glucose consumption, while TMAO treatment increased that of control strain HA1 (EV) cells by 28%, from 12.6 to 16.1 g glucose L^-1^ (Table 4, row 3 vs. row 5), it increased that of transformant YL165-1 cells by much larger magnitude of 66% from 48.0 to 79.9 g glucose L^-1^ (Table 4, row 4 vs. row 6). Accordingly, for cell mass yield, while TMAO treatment increased that of control strain HA1 (EV) cells by 32%, from 3.1 to 4.1 g DCW L^-1^ (Table 4, row 3 vs. row 5), it increased that of transformant YL165-1 by a much larger magnitude 117%, from 10.1 to 21.9 g DCW L^-1^ (Table 4, row 4 vs. row 6).

For lipid production, TMAO treatment increased the total FAME production of control strain HA1 (EV) cells by 11%, from 0.9 to 1.0 g FAME L^-1^ (Table 4, row 3 vs. row 5) and increased that of transformant YL165-1 cells by a much larger magnitude of 74%, from 3.4 to 5.9 g FAME L^-1^ (Table 4, row 4 vs. row 6). In summary, as demonstrated in Table 4, TMAO significantly enhanced glucose consumption and cell mass yield of YL165-1 that led to higher production yield of lipids (i.e., g total lipid per liter). TMAO has been found to stimulate the cell growth of Escherichia coli (Ishimoto and Shimokawa, 1978), *Salmonella typhimurium* (Kim and Chang, 1974) and Proteus sp. strain NTHC153 (Strom et al., 1979), likely by acting as a terminal electron acceptor in the respiratory chain (Strom et al., 1979), thus mediating the cell’s energy metabolism and redox balance. This study observed the stimulation of cell growth of *Y. lipolytica* cellulase-expressing strains; we propose that in addition to the well-documented role of TMAO acting as a chemical chaperon in facilitating protein folding as well as in alleviating ER stress, TMAO may also act as electron acceptor, enhance the cell’s energy metabolism and thus mediate the redox status in the cellulase-expressing *Y. lipolytica* cells. Future studies are needed to explore this complex mechanism. Future studies are also needed to investigate the mechanism of this effect.

For the SFA and UFA profiling, TMAO-treated transformant YL165-1 cells in scenario 3, showed a similar pattern as observed in scenario 2, with significantly more SFA (C16:0 and C18:0) and less UFA (C18:1n9 and C18:2n6; Figure 6C) that leading to a much higher SFA/UFA ratio of 0.74, compared with the TMAO-treated control strain’s SFA/UFA ratio of 0.31 (Figure 6D). Nevertheless, it is noteworthy the SFA/UFA ratio value of 0.74 in scenario 3 was significantly lower that of 1.03 in scenario 2, supporting the literature-reported role of TMAO in reducing the oxidative stress in cells (Sootsuwan et al., 2013; Lamont and Sargent, 2017), which can lead to less oxidative cleavage and less conversion of UFA to SFA.

It is noteworthy that since high C/N ratio medium has been broadly used as a lipid production medium to maximize fatty acids synthesis and lipid accumulation by severely restricting protein synthesis in oleaginous organisms (Back et al., 2016; Yang et al., 2016), the cellulase production of YL165-1 grown in high C/N plus TMAO medium was not assessed in this study. Future studies are needed to investigate any potential effects of TMAO on the cellulase production in engineered *Y. lipolytica* strains.

### Effects of Carbon Source and C/N Ratio on the Fatty Acid Composition Profile of YL165-1

**FIGURE 7:**
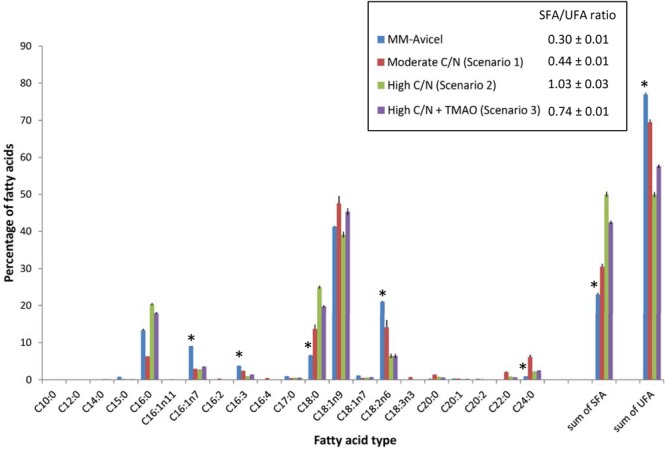
Comparison of fatty acid profiles of *Y. lipolytica* YL165-1 cells grown in four types of media with different carbon sources or C/N ratios. Data shown are the mean from three replicate measurements ± standard error of the mean (SEM). ^∗^ indicates the fatty acids for which MM-Avicel medium-grown cells have different relative levels from other media-grown cells.

The fatty acid profile of YL165-1 cultured in four types of media demonstrates that MM-Avicel medium-grown YL165-1 cells have a unique fatty acid composition different from both the moderate C/N medium, and the high C/N medium (with or without TMAO) (Figure 7). MM-Avicel medium-grown YL165-1 cells have the highest proportion of UFA (C16:1n7, C16:3 and C18:2n6) and lowest SFA (C18:0 and C24:0) than other three media-grown cells (Figure 7). Accordingly, MM-Avicel medium-grown YL165-1 cells have the lowest SFA/UFA ratio. This observation is consistent with a recent study conducted by Dobrowolski et al., which showed that the fatty acid profiles in *Y. lipolytica* strain A101 cultured on various sources of crude glycerol were different, depending on the types of applied substrate (Dobrowolski et al., 2016).

### Factors Affecting the Lipid Contents of *Y. lipolytica* and Their Estimation

It should be noted that since the focus of this study was to co-express fungal cellulases in Yarrowia, we deviated from the optimal culture conditions (such as fermentation in a bioreactor with controlled pH and improved aeration, etc.) used in the previous studies with this strain (Blazeck et al., 2014). As a result, the measured FAME content data for parent control strain HA1 (EV), which was close to 30% FAME DCW basis in shake flasks in this study, cannot be directly compared to the previously reported values, which was in the range of 71% (Liu et al., 2015) to 90% (Blazeck et al., 2014) generated during fermentation in pH controlled, more efficiently aerated bioreactors. Difference between the media and shaking speeds used in flask culture in this vs. previous studies. The previous study used three types of containers (rotary drum, shake flasks, and bioreactor) in assessing lipid accumulation in *Y. lipolytica* strain HA1 (Blazeck et al., 2014). For shake flask culturing of HA1, the different shaking speeds used in the previous study (225 rpm) vs. this study (200 rpm) should partially account for the different levels of lipid accumulation between these two studies as a better agitation/aeration for microbial growth can significantly impact the performance in producing metabolites (Liu et al., 2015). Meanwhile, the different media used in flask culture in this vs. the previous study would likely account for a larger portion of the reported difference in lipid accumulation. In addition to the modified YPD-based medium (YPD-3% Glu that contains yeast extract 10 g L^-1^, peptone 20 g L^-1^, glucose of 30 g L^-1^, which led to a FAME level of 27% ± 2% DCW basis for the strain HA1 cells), this study also used a YNB-based medium that contained 80 g L^-1^ glucose and 1.365 g L^-1^ ammonium (which led to a FAME level of 28% ± 1% DCW basis for the strain HA1 cells); both media were significantly different from the YNB-based medium used in the previous study (Blazeck et al., 2014), which contained up to 160 g L^-1^ glucose and only 0.055 g L^-1^ ammonium and led to a total lipid level up to 90% DCW basis for the strain HA1 cells. The significantly higher C/N ratio of the YNB-based media used in the previous study will likely lead to a higher lipid accumulation for the same strain.

Growth conditions (flask vs. bioreactor). Growth conditions play a significant role in lipid accumulation in *Y. lipolytica*, which is exemplified by the observation of a significant range in lipid accumulation that spans a 74-fold improvement over the parent strain of HA1, strain Po1f, as illustrated in Figure 1 (Blazeck et al., 2014). Another study reported the bioreactor fermentation increased the lipid content of *Y. lipolytica* cells by 50% compared to the shake flask (Tai and Stephanopoulos, 2013).

Buoyancy of *Y. lipolytica* cells. As listed in Table 1, both strains Po1f (the parent strain for HA1 used in the study) and Po1g were derived from the wild-type strain W29 (Madzak et al., 2000). For Po1g, its cell mats were found to float in water after being scrapped off the surface of agar medium, as illustrated in the Supplementary Figure S1 of our previous report (Wei et al., 2014). For HA1, a more recent study showed that a subpopulation of cells had very high buoyancy. The cells floating on top of the medium were full of lipids determined by using fluorescence microscopy with Nile Red staining, whereas normal cells that settled to the bottom of the tube did not contain as much lipids (Liu et al., 2015). In our study, as illustrated in Supplementary Figure S5, the cellulase-co-expressing transformant YL165-1 showed more floating cell mass than the control strain HA1 (EV) in shake flask culture. We speculate that the buoyancy of YL165-1 cells likely also affects the oxygen diffusion into the medium, providing another rationale for optimizing culture conditions by using higher rpm in flask shaking or bioreactor condition to achieve increased lipid accumulation.

### Mechanisms for Relieving the Metabolic Burden of Cellulase Expression by a High C/N Ratio Medium and the Addition of Chemical Chaperone

#### Proposed Role of ER for the Efficient Co-expression of Cellulases and Lipid Biosynthesis

One goal of this study was to examine the dynamic relationship between the production and secretion of heterologous cellulases and the accumulation of lipids. ER is an important organelle for both protein synthesis, folding and secretion, and lipid metabolism. In fact, the synthesis of secretory proteins starts in the ER for correctly integrating nascent proteins and ensuring correct post-translational modification and folding, followed by being captured into ER-derived transport vesicles and delivered to the early Golgi (Barlowe and Miller, 2013; den Haan et al., 2013).

Endoplasmic reticulum also plays a critical role for lipid metabolism, reflected by literature report that (1) a list of 493 candidate proteins (accounting for approximately 9% of the proteome in the yeast *S. cerevisiae*) were known or predicted to be involved in lipid metabolism and its regulation (Natter et al., 2005), and (2) green fluorescent protein (GFP) tagging coupled with confocal laser scanning microscopy was used to localize the above proteins, the majority of tagged, lipid metabolism-related enzymes localized to ER (92), followed by vesicles (53), mitochondria (27), lipid droplets (23), peroxisomes (17), plasma membrane (14), and Golgi (7) (Natter et al., 2005). Furthermore, lipid droplets in yeast are not only functionally connected to the ER (Jacquier et al., 2011), its *de novo* LD biogenesis occurs in ER, and it eventually buds from ER (Jacquier et al., 2013; Choudhary et al., 2015). All these considerations undoubtedly support the ER as the central organelle for protein synthesis, lipid biosynthesis, and lipid droplet formation.

Our results demonstrated that transformant YL165-1 with an overall high level of cellulase secretion compromises the lipid accumulation of these cells. Such an observation, plus the above literature analysis, prompts us to propose an intrinsic link between cellulase co-expression/secretion and lipid accumulation. An enforced overexpression of secreted cellulases will cause a drain on the ER of yeast cells, leading to competition among the co-expressed cellulases for synthesis and secretion, and lipid production.

#### Possible Mechanisms for Relieving the Metabolic Burden by High C/N Ratio Medium and the Addition of Chemical Chaperone

High level expression of heterologous proteins in yeast has been previously found to induce significant cellular changes, including a decrease in growth rate and the altering of nitrogen and redox metabolisms, and poses a metabolic burden on the host cells (Ruijter et al., 2017). In the case of cellulase expression, expression of heterologous *A. aculeatus* and *Saccharomycopsis fibuligera* BGLs had an increasingly negative effect on cell growth of *S. cerevisiae* as the expressed gene doses increased until a final failure to grow (Ding et al., 2017). Equally relevantly, it is reported that high level expression of endogenous and heterologous secreted cellulases can cause ER stress, which subsequently induces the unfolded protein response (UPR), activating related genes to relieve stress in the secretory pathway to improve protein folding efficiency and capacity in filamentous fungi and yeast (Collen et al., 2005; Ilmen et al., 2011; Fan et al., 2015; Van Zyl et al., 2016; Ruijter et al., 2017). The link between ER stress, UPR, and lipid accumulation have been shown in literature that reported ER stress stimulates and increases the level of lipid droplets and protects the yeast cells against the effects of misfolded proteins (Czabany et al., 2008; Fei et al., 2009; Hapala et al., 2011).

The high C/N ratio, limited-nitrogen media have been used to cultivate a range of yeast and algae to achieve high levels of lipid accumulation (Li et al., 2008; Chaisawang et al., 2012; Lamers et al., 2012; Sharma et al., 2012; Braunwald et al., 2013; Sitepu et al., 2013; Blazeck et al., 2014; Hirooka et al., 2014; Zhang et al., 2014; Yu et al., 2015; Yang et al., 2016). As observed in this study, the cellulase co-expression route had an unexpected benefit in enhancing lipid accumulation when grown in medium with high C/N ratio; we propose the likely underlying mechanism would be that the high C/N ratio medium effectively lowers the levels of protein synthesis (because of nitrogen limitation) and ER stress, which leads to boosting nitrogen-limitation-induced lipid production, as shown in Figure 6E (scenario 2). The supplement of TMAO into the high C/N ratio medium likely facilitates the protein folding and lowers the ER and oxidative stresses. TMAO may also help mediate the cell energy metabolism and redox balance via acting as an electron acceptor, as suggested by its enhancement of cell growth and cell mass yield. Future studies are warranted to further explore the mechanisms underlying the delicate balance between the cellulase synthesis/secretion and fatty acid-based biofuels production.

## Conclusion

We have successfully demonstrated the co-expression and secretion of three core fungal cellulases in a high lipid-accumulating engineered strain of the oleaginous yeast, *Y. lipolytica*, enabling nearly a 23% conversion of Avicel. To the best of our knowledge, it represents the first case of exploring the relationship between secreted cellulase expression, cell growth, and lipid production in *Y. lipolytica* strains. The transformant YL165-1 expressed a relatively high titer of EG II and moderate titer of CBH I. Remarkably, when grown in medium with a high C/N ratio and supplemented with a chemical chaperone, the cellulase co-expressing transformant showed a pattern by which the metabolic drain caused by cellulase co-expression and secretion was relieved, and both cell growth and lipid productivity were significantly increased, highlighting the effectiveness of the above approaches in rebalancing the protein synthesis and lipid production in *Y. lipolytica*.

## Author Contributions

MH, MZ, HA, and HW led the project and coordinated the study. HW conceived and designed the experiments. HA provided the lipid hyperaccumulating yeast strains and the genetic background information. HW executed, and WW and QX assisted with DNA construct building, yeast transformation and screening, SDS–PAGE, western blotting analysis, and enzymatic analyses. SVW conducted the FAME analysis. C-YL, YL, and HW designed the primers and conducted the RNA extraction and real-time RT-PCR. SD provided CBH I, CBH II and EG II antibodies, and provided guidance on enzymatic assays. EK provided guidance on yeast culturing techniques and media. HW prepared the initial draft of the manuscript. SD, EK, MH, HA, and MZ edited the manuscript. HW coordinated the manuscript submission. All authors read and approved the final manuscript.

## Conflict of Interest Statement

The authors declare that the research was conducted in the absence of any commercial or financial relationships that could be construed as a potential conflict of interest.

## Abbreviations

BGL: β-glucosidase
CBH: cellobiohydrolase
DCW: dry cell weight
DGA: diacylglycerolacyltransferase
ER: endoplasmic reticulum
FAME: fatty acid methyl esters
hp4d: hybrid promoter derived from pXPR2
Te: Talaromyces emersonii
TMAO: trimethylamine N-oxide dihydride
Tr: *Trichoderma reesei*
Te-Tr: Talaromyces emersonii-*Trichoderma reesei*
XPR2: alkaline extracellular protease 2
YP: yeast extract-peptone (medium)
YPD: yeast extract-peptone-dextrose (medium)
