# Supplementary material for: Ameliorating the Metabolic Burden of the Co-expression of Secreted Fungal Cellulases in a High Lipid-Accumulating Yarrowia lipolytica Strain by Medium C/N Ratio and a Chemical Chaperone
Source: Front Microbiol. 2019 Jan 9;9:3276. doi: 10.3389/fmicb.2018.03276 (PMC6333634; doi:10.3389/fmicb.2018.03276)
Supplement: Supplementary file 1 [file Data_Sheet_1.pdf]

Ameliorating the metabolic burden of the co-expression of secreted fungal cellulases in a high lipid-accumulating *Yarrowia lipolytica* strain by medium C/N ratio and a chemical chaperone

Additional file 1:

File type: .pdf

List of contents: **Page no.**

Supplementary Materials and Methods 2

Supplementary Table S1. Primer sequences for real-time RT PCR 5

Supplementary Figure S1. Growth of *Y. lipolytica* transformants for co-expressing CBH I, CBH II, and EG II on PASC-YPD agar plate 6

Supplementary Figure S2. Transcript levels relative to YL165-1 for CBH I-CBH II-EG II co-expressed in *Y. lipolytica* transformants YL165-1, -5, -6, -7, and -8. 7

Supplementary Figure S3. Effects of concentrated crude enzymes from transformant YL165-1 on the morphology of wet ball-dispersed Avicel particles 8

Supplementary Figure S4: The timeline profiling of cell mass weight of *Y. lipolytica* transformants expressing multiple cellulases and the control strain HA1 (EV) in shake flasks. 9

Supplementary Figure S5. Floating cell mats of *Y. lipolytica* HA1 (EV) and transformant YL165-1. 10

Supplementary Materials and Methods

Nucleotide sequences of the cellulase expression cassesttes of construct 162.

Note that the nucleotide sequence ((length: 7780 bp) was codon-optimized based on the codon bias of *Y. lipolytica*, with the SalI site (GTCGAC) and PmlI site (CACGTG) at the 5’ end, and the KpnI site (GGTACC) at the 3’ end for cloning into the vector pUC57 and pYLEX (see the Methods section for details).

>pNREL162_construct_CBH I-CBH II-EG II
GTCGACCACGTGAGAGACCGGGTTGGCGGCGCATTTGTGTCCCAAAAAACAGCCCCAATTGCCCCAAT
TGACCCCAAATTGACCCAGTAGCGGGCCCAACCCCGGCGAGAGCCCCCTTCTCCCCACATATCAAACC
TCCCCCGGTTCCCACACTTGCCGTTAAGGGCGTAGGGTACTGCAGTCTGGAATCTACGCTTGTTCAGAC
TTTGTACTAGTTTCTTTGTCTGGCCATCCGGGTAACCCATGCCGGACGCAAAATAGACTACTGAAAATT
TTTTTGCTTTGTGGTTGGGACTTTAGCCAAGGGTATAAAAGACCACCGTCCCCGAATTACCTTTCCTCT
TCTTTTCTCTCTCTCCTTGTCAACTCACACCCGAAATCGTTAAGCATTTCCTTCTGAGTATAAGAATCAT
TCAAAATGGTGAGTTTCAGAGGCAGCAGCAATTGCCACGGGCTTTGAGCACACGGCCGGGTGTGGTCC
CATTCCCATCGACACAAGACGCCACGTCATCCGACCAGCACTTTTTGCAGTACTAACCGCAGAAGCTC
GCTACCGCCTTTACTATTCTCACGGCCGTTCTGGCCCAGCAGGCAGGGACCGCTACCGCCGAGAACCA
TCCTCCCCTCACCTGGCAGGAGTGCACCGCACCCGGATCCTGCACCACGCAGAACGGCGCTGTCGTGC
TGGACGCAAATTGGCGATGGGTTCACGATGTGAACGGGTACACTAACTGCTATACAGGCAACACATGG
GATCCTACCTACTGCCCCGACGACGAAACATGTGCCCAGAACTGTGCCCTTGACGGAGCTGACTACGA
GGGAACCTACGGGGTCACCTCGTCCGGGAGCAGTCTCAAGCTCAACTTTGTGACCGGTTCAAACGTCG
GTTCACGGCTCTATCTGCTCCAGGACGACTCGACCTACCAGATCTTCAAACTGTTGAACCGAGAGTTCT
CGTTTGACGTTGATGTTTCGAACTTGCCTTGCGGACTTAATGGTGCTCTGTACTTTGTTGCAATGGATG
CTGACGGCGGAGTCTCTAAGTACCCCAACAACAAGGCTGGTGCCAAGTACGGTACGGGCTATTGTGAC
AGTCAGTGCCCTAGAGATCTAAAATTCATTGATGGCGAGGCCAACGTCGAGGGCTGGCAACCGAGCA
GCAATAACGCCAATACTGGAATCGGCGACCACGGCTCCTGTTGCGCGGAGATGGACGTGTGGGAAGC
GAACTCCATTAGTAATGCTGTAACACCCCATCCGTGCGACACTCCGGGACAGACGATGTGTTCCGGTG
ACGATTGTGGCGGCACCTACTCCAACGATCGATACGCGGGTACATGCGATCCCGACGGCTGCGATTTC
AACCCATACCGGATGGGTAATACATCCTTCTATGGACCAGGTAAGATTATCGACACTACGAAGCCTTT
CACCGTGGTCACGCAGTTTTTGACAGATGACGGCACTGATACCGGAACCCTTTCGGAAATCAAGCGCT
TTTACATCCAGAACTCTAATGTTATTCCCCAACCCAACAGTGACATTTCCGGAGTTACTGGAAACTCTA
TCACTACAGAGTTTTGTACAGCCCAAAAGCAGGCCTTCGGGGACACCGACGACTTTTCTCAGCACGGA
GGCCTGGCCAAAATGGGAGCTGCCATGCAACAGGGAATGGTGCTTGTCATGTCTCTGTGGGATGACTA
CGCCGCTCAAATGCTGTGGCTGGACTCTGACTACCCCACTGATGCCGATCCAACTACCCCTGGCATCGC
CAGAGGTACTTGTCCCACCGACAGCGGCGTGCCGTCCGACGTAGAGTCCCAGTCGCCCAACAGCTATG
TGACGTACTCGAACATTAAGTTCGGCCCTATCAACTCTACGTTCACCGCATCTAACCCACCTGGTGGTA
ACCGAGGTACTACGACCACTCGACGTCCTGCTACTACCACTGGTTCGTCACCCGGACCCACCCAGTCTC
ACTACGGACAGTGTGGCGGAATTGGATACTCTGGTCCCACCGTGTGTGCGTCTGGAACTACCTGTCAG
GTCCTCAACCCTTACTACTCCCAATGCCTGTAATAGGCAATTAACAGATAGTTTGCCGGTGATAATTCT
CTTAACCTCCCACACTCCTTTGACATAACGATTTATGTAACGAAACTGAAATTTGACCAGATATTGTTG
TAAATAGAAAATCTGGCTTGTAGGTGGCAAAATGCGGCGTCTTTGTTCATCAATTCCCTCTGTGACTAC
TCGTCATCCCTTTATGTTCGACTGTCGTATTTCTTATTTTCCATACATATGCAAGTGAGATGCCCGTGTC
CGAATTCGACGCAGTAGGATGTCCTGCACGGGTCTTTTTGTGGGGTGTGGAGAAAGGGGTGCTTGGAG
ATGGAAGCCGGTAGAACCGGGCTGCTTGTGCTTGGAGATGGAAGCCGGTAGAACCGGGCTGCTTGGG
GGGATTTGGGGCCGCTGGGCTCCAAAGAGGGGTAGGCATTTCGTTGGGGTTACGTAATTGCGGCATTT
GGGTCCTGCGCGCATGTCCCATTGGTCAGAATTAGTCCGGATAGGAGACTTATCAGCCAATCACAGCG
CCGGATCCACCTGTAGGTTGGGTTGGGTGGGAGCACCCCTCCACAGAGTAGAGTCAAACAGCAGCAG
CAACATGATAGTTGGGGGTGTGCGTGTTAAAGGAAAAAAAAGAAGCTTGGGTTATATTCCCGCTCTAT
TTAGAGGTTGCGGGATAGACGCCGACGGAGGGCAATGGCGCCATGGAACCTTGCGGATATCGATACG
CCGCGGCGGACTGCGTCCGAACCAGCTCCAGCAGCGTTTTTCCGGGCCATTGAGCCGACTGCGACCC
CGCCAACGTGTCTTGGCCCACGCACTCATGTCATGTTGGTGTTGGGAGGCCACTTTTTAAGTAGCACAA
GGCACCTAGCTCGCAGCAAGGTGTCCGAACCAAAGAAGCGGCTGCAGTGGTGCAAACGGGGCGGAAA
CGGCGGGAAAAAGCCACGGGGGCACGAATTGAGGCACGCCCTCGAATTTGAGACGAGTCACGGCCCC
ATTCGCCCGCGCAATGGCTCGCCAACGCCCGGTCTTTTGCACCACATCAGGTTACCCCAAGCCAAACC
TTTGTGTTAAAAAGCTTAACATATTATACCGAACGTAGGTTTGGGCGGGCTTGCTCCGTCTGTCCAAGG

CAACATTTATATAAGGGTCTGCATCGCCGGCTCAATTGAATCTTTTTTCTTCTTCTCTTCTCTATATTCA
TTCTTGAATTAAACACACATCAACATGAAGCTCGCTACCGCCTTTACTATTCTCACGGCCGTTCTGGCC
CAGGCGTGCAGTAGTGTTTGGGGCCAGTGCGGTGGTCAAAACTGGTCTGGCCCGACTTGCTGTGCTTC
TGGTTCGACCTGTGTTTACTCGAACGATTACTACTCTCAATGCCTGCCGGGAGCTGCCAGCTCCAGTTC
ATCCACGAGAGCCGCGTCTACAACATCACGAGTGTCCCCTACGACCTCTCGCTCATCGAGCGCAACTC
CTCCCCCTGGGTCTACCACAACCCGGGTCCCACCTGTGGGATCGGGAACTGCCACGTACTCCGGCAAT
CCTTTTGTGGGCGTGACACCTTGGGCAAACGCCTACTACGCTAGTGAAGTGTCGTCGCTGGCCATTCCC
TCTCTCACTGGAGCTATGGCAACGGCTGCCGCTGCCGTCGCAAAGGTCCCCTCCTTCATGTGGCTGGAC
ACTCTTGATAAGACCCCACTCATGGAGCAAACTCTGGCAGACATTCGAACTGCGAACAAAAACGGTGG
TAACTACGCCGGACAGTTCGTGGTCTATGACCTTCCCGACAGAGACTGCGCTGCCCTGGCCTCTAACG
GCGAATATTCTATTGCGGATGGAGGGGTCGCCAAGTACAAAAACTATATTGACACGATCCGGCAGATC
GTCGTTGAGTACTCTGACATCCGTACCCTCCTCGTCATCGAGCCCGATTCCCTTGCCAACTTGGTTACC
AACCTGGGTACACCTAAGTGTGCAAACGCCCAGTCTGCTTACCTGGAGTGTATTAACTATGCTGTTACC
CAACTGAACCTCCCCAATGTCGCCATGTACCTCGATGCTGGACACGCCGGTTGGCTGGGGTGGCCTGC
TAATCAGGACCCCGCTGCTCAGCTTTTCGCTAATGTATACAAAAACGCTTCCTCCCCCAGGGCCCTTCG
AGGACTCGCCACTAACGTTGCCAACTACAACGGTTGGAACATTACCTCTCCCCCATCCTACACCCAGG
GTAACGCAGTGTATAACGAGAAGCTGTACATCCATGCTATCGGACCCCTGCTCGCCAATCATGGATGG
AGCAACGCCTTTTTCATCACCGACCAGGGCCGATCGGGCAAGCAGCCCACCGGCCAGCAGCAGTGGG
GCGATTGGTGTAATGTGATCGGAACCGGTTTCGGTATTCGTCCATCTGCCAATACAGGCGATTCCCTGC
TCGACTCATTTGTGTGGGTCAAGCCTGGCGGAGAGTGTGACGGCACCTCGGACAGCTCCGCTCCGCGA
TTCGATAGCCACTGCGCACTGCCGGACGCTTTGCAGCCCGCTCCTCAGGCCGGAGCGTGGTTCCAAGC
CTACTTTGTGCAGCTATTGACTAACGCCAACCCCTCGTTTCTATGATAAGCTATTTATCACTCTTTACAA
CTTCTACCTCAACTATCTACTTTAATAAATGAATATCGTTTATTCTCTATGATTACTGTATATGCGTTCC
TCTAAGACAAATCGAAACCAGCATGCGATCGAATGGCATACAAAAGTTTCTTCCGAAGTTGATCAATG
TCCTGATAGTCAGGCAGCTTGAGAAGATTGACACAGGTGGAGGCCGTAGGGAACCGATCAACCTGTCT
ACCAGCGTTACGAATGGCAAATGACGGGTTCAAAGCCTTGAATCCTTGCAATGGTGCCTTGGATACTG
ATGTCACAAACTTAAGAAGCAGCCGCTTGTCCTCTTCCTCGAAACTGAGTTTGGCGCCCGTTTTTTCGA
GCCCCACACGTTTCGGTGAGTATGAGCGGCGGCAGATTCGAGCGTTTCCGGTTTCCGCGGCTGGACGA
GAGCCCATGATGGGGGCTCCCACCACCAGCAATCAGGGCCCTGATTACACACCCACCTGTAATGTCAT
GCTGTTCATCGTGGTTAATGCTGCTGTGTGCTGTGTGTGTGTGTTGTTTGGCGCTCATTGTTGCGTTATG
CAGCGTACACCACAATATTGGAAGCTTATTAGCCTTTCTATTTTTTCGTTTGCAAGGCTTAACAACATT
GCTGTGGAGAGGGATGGGGATATGGAGGCCGCTGGAGGGAGTCGGAGAGGCGTTTTGGAGCGGCTTG
GCCTGGCGCCCAGCTCGCGAAACGCACCTAGGACCCTTTGGCACGCCGAAATGTGCCACTTTTCAGTC
TAGTAACGCCTTACCTACGTCATTCCATGCATGCATGTTTGCGCCTTTTTTCCCTTGCCCTTGATCGCCA
CACAGTACAGTGCACTGTACAGTGGAGGTTTTGGGGGGGTCTTAGATGGGAGCTAAAAGCGGCCTAGC
GGTACACTAGTGGGATTGTATGGAGTGGCATGGAGCCTAGGTGGAGCCTGACAGGACGCACGACCGG
CTAGCCCGTGACAGACGATGGGTGGCTCCTGTTGTCCACCGCGTACAAATGTTTGGGCCAAAGTCTTG
TCAGCCTTGCTTGCGAACCTAATTCCCAATTTTGTCACTTCGCACCCCCATTGATCGAGCCCTAACCCC
TGCCCATCAGGCAATCCAATTAAGCTCGCATTGTCTGCCTTGTTTAGTTTGGCTCCTGCCCGTTTCGGC
GTCCACTTGCACAAACACAAACAAGCATTATATATAAGGCTCGTCTCTCCCTCCCAACCACACTCACTT
TTTTGCCCGTCTTCCCTTGCTAACACAAAAGTCAAGAACACAAACAACCACCCCAACCCCCTTACACA
CAAGACATATCTACAGCAATGAAGCTCGCTACCGCCTTTACTATTCTCACGGCCGTTCTGGCCCAACAG
ACGGTGTGGGGCCAATGTGGCGGCATTGGCTGGTCGGGTCCGACCAACTGTGCTCCTGGCTCGGCCTG
TTCGACTCTGAACCCCTACTACGCTCAGTGTATCCCCGGAGCGACCACCATCACCACGTCTACTCGACC
CCCTTCAGGACCCACTACCACCACGCGAGCGACCTCCACCTCCAGCTCTACTCCCCCTACATCCTCTGG
TGTTCGATTTGCTGGCGTGAACATCGCCGGTTTCGACTTCGGATGCACGACTGACGGTACATGCGTGAC
ATCCAAGGTCTATCCCCCGTTGAAAAACTTCACCGGGTCTAACAATTATCCTGACGGCATTGGTCAGAT
GCAACACTTTGTCAACGAGGATGGCATGACCATCTTCCGACTCCCTGTCGGCTGGCAGTATCTAGTGA
ACAATAACCTGGGCGGAAACCTTGACAGCACAAGCATCTCCAAGTACGATCAATTGGTGCAGGGATG
CCTCAGTTTGGGTGCTTACTGCATCGTTGACATTCACAACTACGCTAGGTGGAACGGGGGAATCATCG
GACAGGGTGGCCCCACTAACGCCCAGTTCACAAGTCTGTGGTCCCAGCTCGCCTCTAAGTACGCATCT
CAGTCCCGGGTGTGGTTTGGAATCATGAATGAGCCCCATGACGTCAACATTAACACTTGGGCTGCCAC
CGTACAGGAAGTCGTTACCGCCATTCGTAACGCCGGAGCCACCTCGCAGTTCATCTCGCTGCCTGGAA
ATGACTGGCAGTCTGCTGGTGCTTTCATTTCCGATGGATCAGCAGCCGCTCTCAGCCAAGTCACGAACC
CTGATGGATCTACAACCAACCTGATTTTCGACGTGCATAAGTACCTGGACTCGGATAACAGCGGAACC
CACGCGGAGTGTACCACTAACAACATTGACGGTGCCTTTTCTCCTCTCGCAACGTGGCTCAGACAGAA

CAACAGACAGGCCATTCTGACTGAAACTGGTGGCGGCAACGTTCAATCATGTATCCAGGACATGTGCC
AGCAGATTCAGTACCTGAATCAGAATAGTGACGTGTACCTTGGCTACGTGGGTTGGGGAGCAGGGTCC
TTTGATTCTACCTACGTCCTGACCGAGACTCCGACATCCTCCGGCAACTCGTGGACTGATACATCTCTT
GTTTCGTCATGCCTTGCCCGCAAGTGATAAAGCTTTGCGGCGAAACTCGATTCTCACCCTCGATAACTC
GACTCACCCCCCTTAACTTAAAATTCACTTACGACAAACAAACGTCTGATACCGACTACCCCCTCGACT
TCTCGCAATCTCGACTTTCAATCAGAGGACCTCAAACAACCACTTTTTTCTTACGATTCTAATTATTTAC
CCATTCATTAATTTCCCGTGCGCTCGTCCAGCAATGTCCGAGAGCATCCTGCCTGTGTCTCTGGGCCCA
TCCATTTAATTTGGGTCCATCCTCCCGGGCAGATTCCACAGTCCAGTTGTCGCCGACTGGATGGTTAGT
AGATCCGCCTTTTTTAGTTTGAACATGTTGCGAGTTGATACCTGAACATCAGAGTTTTAGCTTCTTGTA
CTAATATACATTCCTTTTAGTGAGGTCCTGTACACTGTACACTGTATTATATTGTAGCGTTGTATTTTTG
ATCCCTTGGTTTGTTCGTTATACGGTGTACAAGTATGTACGATCGGTACC,

Supplementary Table S1. Primer sequences for real-time RT PCR.

Target genes	Primer sequences	Amplicon size (bp)
Actin	Yl-Actin-1-F: TCCAGGCCGTCCTCTCCC<br>Yl-Actin-1-R: GGCCAGCCATATCGAGTCGCA	141
chimeric CBH I	CBH I-2-F: TTCACCGCATCTAACCCACC<br>CBH I-2-R: GGTAGTTCCAGACGCACACA	159
CBH II	CBH II-1-F: GACCCCACTCATGGAGCAAA<br>CBH II-1-R: TTTGTACTTGGCGACCCCTC	166
EG II	EG II-3-F: TGACCATCTTCCGACTCCCT<br>EG II-3-R: ACGATGCAGTAAGCACCCAA	136

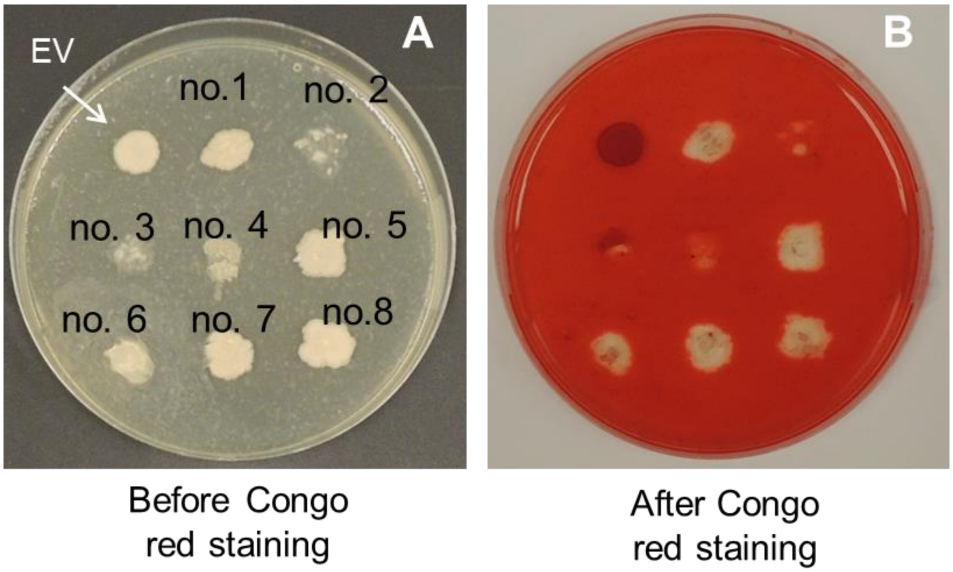

Supplementary Figure S1. Growth of *Y. lipolytica* transformants co-expressing CBH I, CBH II, and EG II on PASC-YPD agar plate. Eight transformants were grown on PASC-YPD agar plate (6 d) before (**A**) and after the Congo Red staining (**B**). EV, parent strain transformed with empty vector.

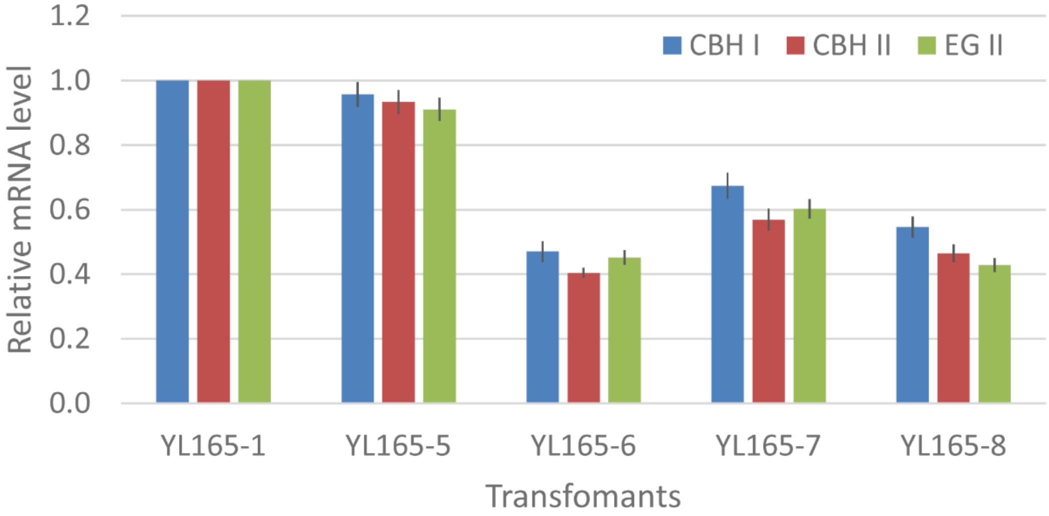

Supplementary Figure S2. Transcript levels relative to YL165-1 for CBH I-CBH II-EG II co-expressed in *Y. lipolytica* transformants YL165-1, -5, -6, -7, and -8. Data from three biological replicates, with error bars indicating the standard error of the mean (SEM).

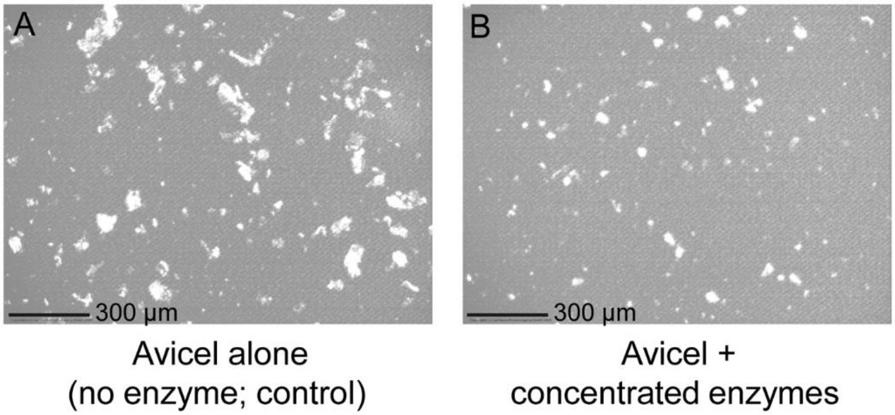

Supplementary Figure S3. Effects of concentrated crude enzymes from transformant YL165-1 on the morphology of wet ball-dispersed Avicel particles. Morphological comparison of wet ball-dispersed Avicel granules after 5 d incubation with no enzymes **(A)** versus with 35x concentrated crude enzymes of *Y. lipolytica* strain YL165-1 **(B)**. The scale bar represents 300 µm.

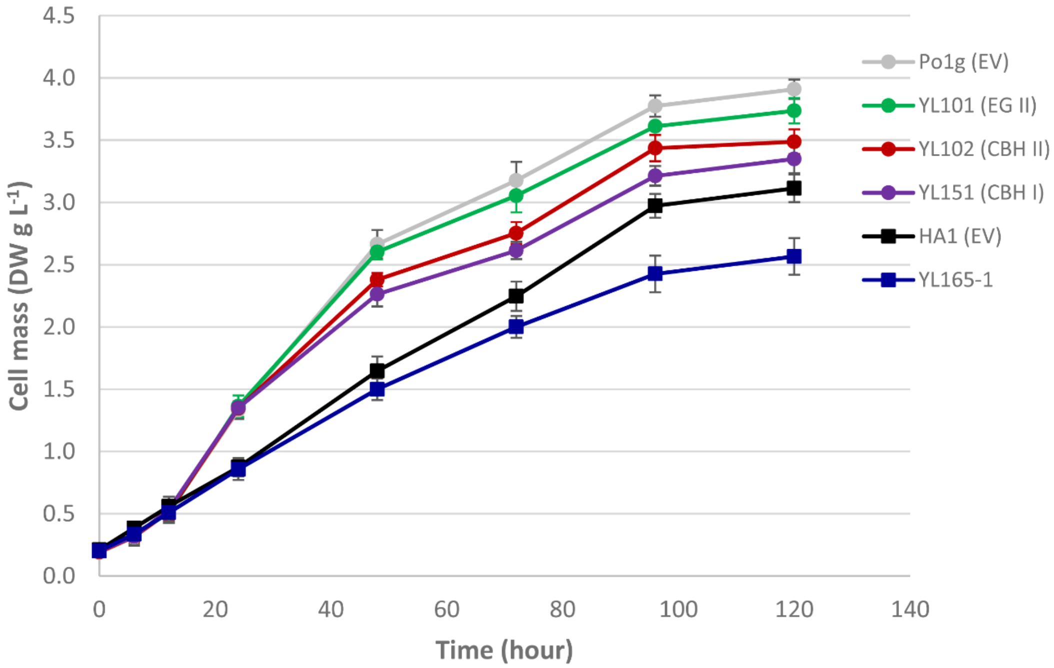

Supplementary Figure S4: The timeline profiling of cell mass weight of *Y. lipolytica* transformants expressing multiple cellulases and the control strain HA1 (EV) in shake flasks.

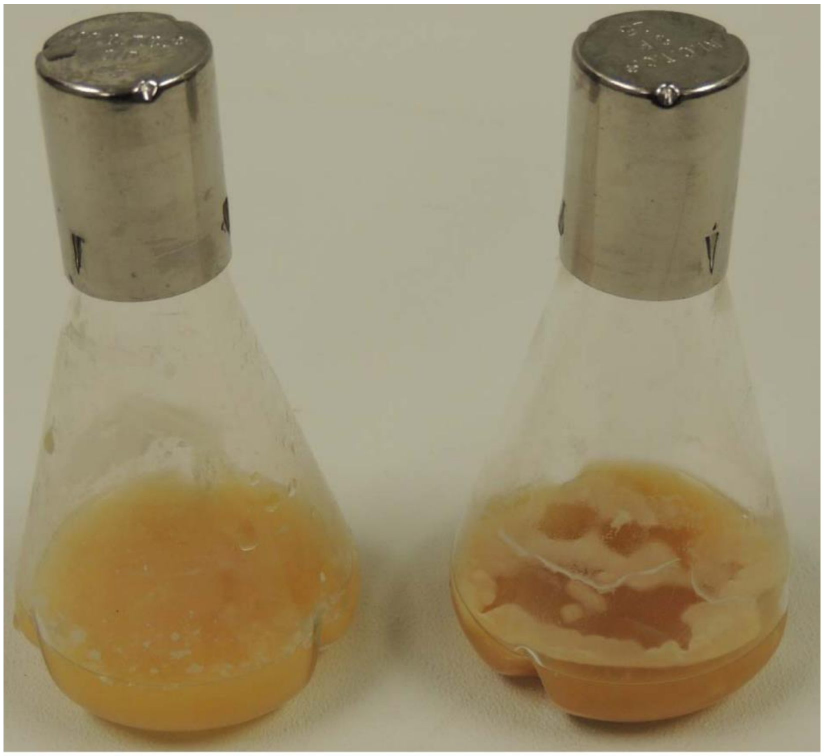

HA1 (EV) YL165-1

Supplementary Figure S5. Floating cell mats of *Y. lipolytica* HA1 (EV) and transformant YL165-1. The cells were cultured in YPD-3% glucose medium in 250-mL baffled flasks at 200 rpm, 28°C for 6 d.
